# Toward Sustainable Wearable Electronic Textiles

**DOI:** 10.1021/acsnano.2c07723

**Published:** 2022-11-30

**Authors:** Marzia Dulal, Shaila Afroj, Jaewan Ahn, Yujang Cho, Chris Carr, Il-Doo Kim, Nazmul Karim

**Affiliations:** † Centre for Print Research (CFPR), University of the West of England, Frenchay Campus, BristolBS16 1QY, United Kingdom; ‡ Department of Materials Science and Engineering, Korea Advanced Institute of Science and Technology (KAIST), Daejeon34141, Republic of Korea; § Clothworkers’ Centre for Textile Materials Innovation for Healthcare, School of Design, University of Leeds, LeedsLS2 9JT, United Kingdom

**Keywords:** wearable electronics, e-textiles, sustainability, biodegradability, recyclability, sustainable electronics, smart textiles, life cycle analysis

## Abstract

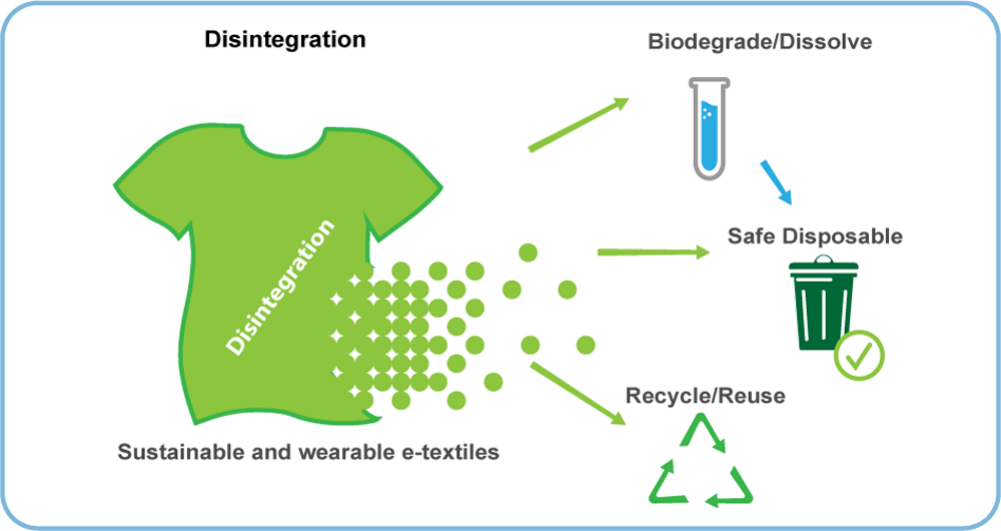

Smart wearable electronic textiles (e-textiles) that can detect and differentiate multiple stimuli, while also collecting and storing the diverse array of data signals using highly innovative, multifunctional, and intelligent garments, are of great value for personalized healthcare applications. However, material performance and sustainability, complicated and difficult e-textile fabrication methods, and their limited end-of-life processability are major challenges to wide adoption of e-textiles. In this review, we explore the potential for sustainable materials, manufacturing techniques, and their end-of-the-life processes for developing eco-friendly e-textiles. In addition, we survey the current state-of-the-art for sustainable fibers and electronic materials (i.e., conductors, semiconductors, and dielectrics) to serve as different components in wearable e-textiles and then provide an overview of environmentally friendly digital manufacturing techniques for such textiles which involve less or no water utilization, combined with a reduction in both material waste and energy consumption. Furthermore, standardized parameters for evaluating the sustainability of e-textiles are established, such as life cycle analysis, biodegradability, and recyclability. Finally, we discuss the current development trends, as well as the future research directions for wearable e-textiles which include an integrated product design approach based on the use of eco-friendly materials, the development of sustainable manufacturing processes, and an effective end-of-the-life strategy to manufacture next generation smart and sustainable wearable e-textiles that can be either recycled to value-added products or decomposed in the landfill without any negative environmental impacts.

Smart wearable electronic textiles (e-textiles) are becoming increasingly attractive due to their intriguing electrical, thermal, and optical functionalities, as well as being able to collect physiological data in real-time for continuous health monitoring applications.^1−4^ Indeed, what makes smart wearable e-textiles exciting is their potential for developing highly innovative and intelligent clothing that can function as sensors, heaters, energy generating and storage devices simultaneously.^1^ Multifunctional wearable e-textiles that can detect and distinguish multiple different types of stimuli, while also collecting and storing a wide range of signals all using just a single device, are of high value for personalized healthcare applications.^5−7^ However, the significant barriers for wide adoption of e-textiles are poor material performance and sustainability, complicated and time-consuming fabrication methods, numerous toxic wastes generated from manufacturing processes, and their limited end-of-life portion processability.^8−11^

With an estimated 92 million tonnes of textile waste produced annually,^12−14^ the textile industry is thought to be the second largest environmental polluter behind the oil sector,^15,16^ with an equivalent to a garbage truck full of clothing being dumped into the ground every second.^17^ Although 95% of textiles are fully recyclable,^18^ ∼85% of all textiles are still dumped into landfills.^17,19,20^ The incorporation of electronics into existing textiles to produce e-textiles will therefore make end-of-life processing even more complicated and challenging.^10^ Indeed, wearable e-textiles containing nontextile components, such as electronics, batteries and interconnections, are challenging or even impractical to dissemble. For example, current electronics can comprise of up to 57 types of components,^21^ each of which requires specific recycling methods due to their varying physical/chemical properties. Many components are valuable, while others are hazardous heavy metals and halogenated organic compounds that can impact individual health and generate environmental risks.^22,23^ Therefore, there is a clear unmet need for an integrated product design, the use of eco-friendly materials, the development of sustainable manufacturing processes, and an effective end-of-the-life strategy to encourage the manufacture of the next generation of smart and sustainable wearable e-textiles that can either be recycled to value-added products or decomposed in the landfill without any negative environmental impacts.

Here, we review the potential of sustainable materials and manufacturing techniques for developing eco-friendly wearable e-textiles that can either be decomposed or recycled after use. Within the discussion, we examine the nature of sustainable fibers including biosynthetic and recycled fibers, the modulation of their electrical properties using sustainable electronic materials (i.e., conductors, semiconductors, and dielectrics) to serve as different components in wearable e-textiles, and the potential for innovative manufacturing techniques that facilitate sustainable production plants based on waterless technologies and lower energy consumption. In addition, standardized parameters for evaluating the sustainability of e-textiles are established, such as life cycle analysis (LCA), biodegradability, and recyclability. Finally, the current development trends, as well as future research directions for wearable e-textiles are identified.

## Sustainable Materials

### Sustainable Fiber Materials

The major component in smart wearable e-textiles are either natural (e.g., cotton, flax) or man-made (e.g., polyester, acrylic) fibers.^24^ Polyester is the most widely used textile fiber, comprising ∼51% (∼54 million tonnes) of total consumption. Cotton is the second most widely used fibers at 25% (∼26 million tonnes) in global consumption.^12^ Although cotton is a naturally produced cellulosic fiber material that is also biodegradable,^25^ the manufacturing of conventional cotton fibers is regarded as environmentally unfriendly^26^ and involves significant water consumption.^27^ In general, a significant amount of water (>20,000 L/kg of fibers) is required to process cotton textiles,^28^ which has significant environmental impacts on most cotton-based textiles producing countries such as Bangladesh, India, and China, where there is already a shortage of fresh drinking water.^29^ Additionally, cotton fibers consume ∼10% and 25% of the global pesticides and insecticides,^30^ respectively, with a large portion^31^ of the pesticides being discarded as runoff into rivers^32^ or the soil,^33^ which has become a threat to the environment and marine life.^32^ Nevertheless, man-made cellulosic fibers (such as viscose, modal, and lyocell) could potentially play a significant role in realizing sustainable and circular fashion by becoming carbon sinks and enhancing public resilience and benefits.^34,35^

Polyester, the most commonly used textile fiber, is man-made and derived from nonrenewable fossil fuels. The raw materials for polyester fibers, such as polyethylene terephthalate (PET), are produced by converting crude oil into petrochemicals, during which toxins such as ethylene, propylene, butadiene, and methanol are released into the atmosphere^36^ and pose severe risks to human life and the ecosystem at large.^37^ Additionally, polyester fibers are not biodegradable, and their production process is also a highly energy-intensive. For example, ∼125 MJ of energy is required to produce 1 kg of polyester fiber, with a staggering CO_2_ emission level of 14.2 kg/kg_._^38,39^ Considering the finite supply of resources on Earth, the rapidly growing global population, and the shrinkage of arable land available for cultivation, there is a clear need for an alternative approach to textile manufacturing based on sustainability.^40,41^

**Figure 1:**
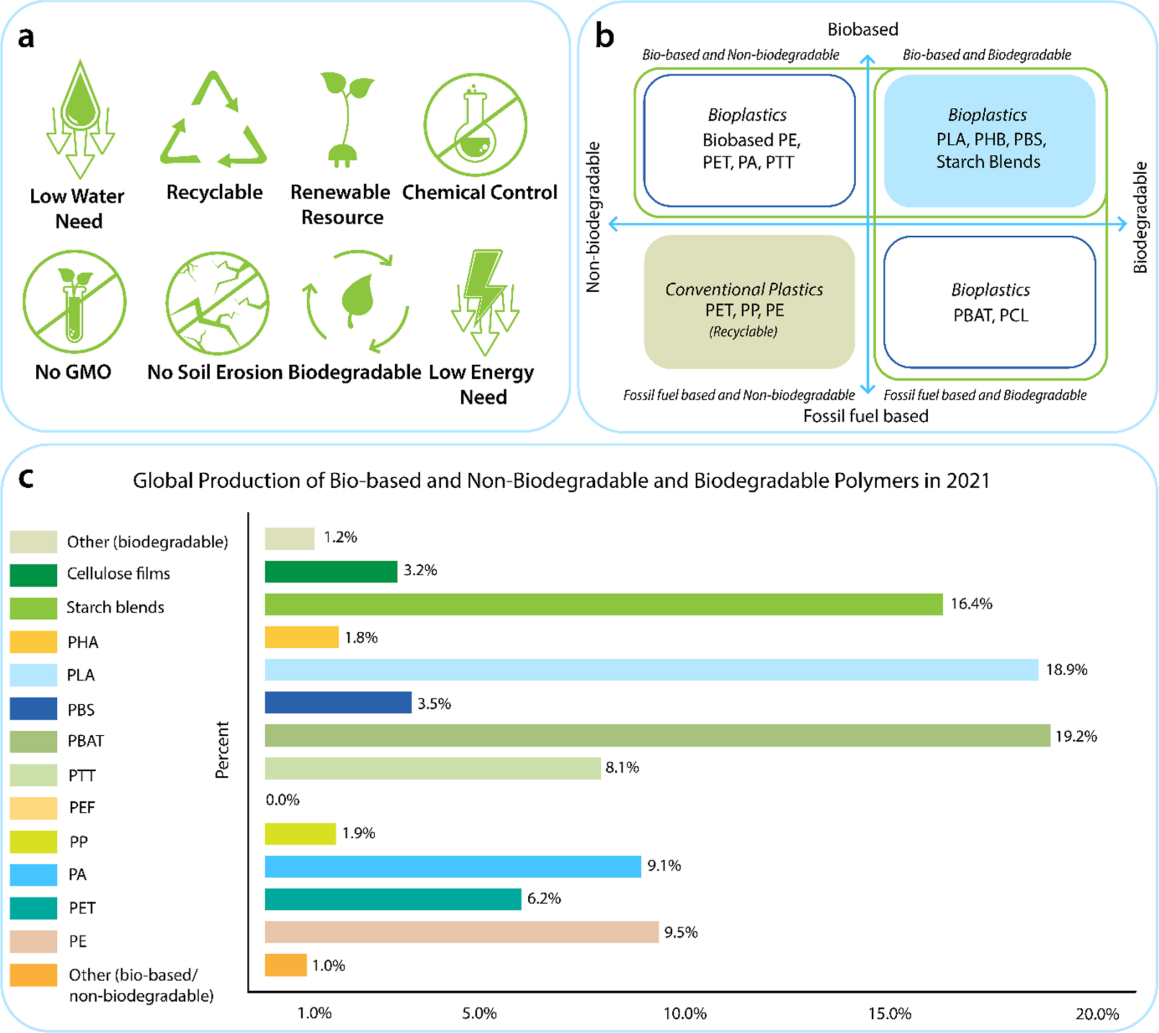
(a) Criteria for sustainable fibers. (b) Classification of plastic. (c) Global production of bio-based non-biodegradable and biodegradable polymers in 2021.^43^

Sustainability is a multifaceted concept involving the integration of better usage of materials, reducing the overall carbon footprints, and balancing the use of renewable resources and biodegradable products with an efficient recycling/remanufacturing economy. Through this holistic approach, a global solution to fiber production, textile manufacturing, and sustainability can be achieved, and the main factors are identified in Figure 1a. The development of sustainable fibrous polymers will enable conservation of limited natural resources and provide a potential solution to the key challenges generated by plastic-based synthetic polymers. Various research strategies have been previously identified which will contribute toward developing an integrated strategy.^42^

Biopolymers have emerged as a latest class of sustainable and natural^44^ alternatives to current fossil-fuel-based fibers.^45^ “Bio-based” refers to the origin of the material which is either partly or totally bio-based. Biopolymers are derived from renewable resources, typically plants or plant-derived matter (e.g., sugar cane, cassava, corn). Note that the label “bio-based” only indicates the source of the materials and does not address implications about its end-of-life. In contrast, biodegradable plastics undergo chemical processes for their conversion into natural materials (e.g., carbon dioxide, biomass, and water) with the aid of microbes that are present in the environment. The environmental conditions must be optimal for biodegradation to occur efficiently. Though not all bio-based plastics are biodegradable, some exhibit biodegradable characteristics, together physical properties that are not dissimilar from petroleum-based plastics.

Examples of bio-based and biodegradable bioplastics include starch-derived polylactic acid (PLA), polyhydroxyalkanoates (PHA), microbe-derived polyhydroxybutyrate (PHB), and polybutylene succinate (PBS).^46^ However, most of the current bio-based plastics are non-biodegradable, which causes waste management problems.^47^ For instance, bio-based “drop-in” polyethylene (PE), polypropylene (PP), PET, thermoplastic polyester elastomers (TPC-ET), and bio-based polyamides (PA) are all non-biodegradable bioplastics derived from renewable natural resources.^48^ In contrast, some fossil-fuel-based plastics such as polybutylene adipate terephthalate (PBAT) and polycaprolactone (PCL) exhibit biodegradable characteristics despite not originating from bio-based sources.^49−51^ PBAT is fully biodegradable when composted due to the presence of butylene adipate groups. However, it should be noted that the terephthalate chain sections, which provide the material’s high stability and mechanical properties, do not degrade in marine and fresh water. In addition, while some plastics (fossil-fuel-based and non-biodegradable) may be recyclable to some extent,^52^ after a finite number of cycles they eventually end up in the landfill as trash.^53,54^ Therefore, the selection of the “best” sustainable fibrous materials, as listed in Figure 1b, provides an opportunity to establish a long-term strategy for the design of sustainable and multifunctional wearable e-textiles but also for the betterment of the environment, society, and the global economy. Thus, plastic polymers can be classified into four groups and the global production data,^43^ shown in Figure 1c, highlights the significant production of PBAT in 2021.

**Figure 2:**
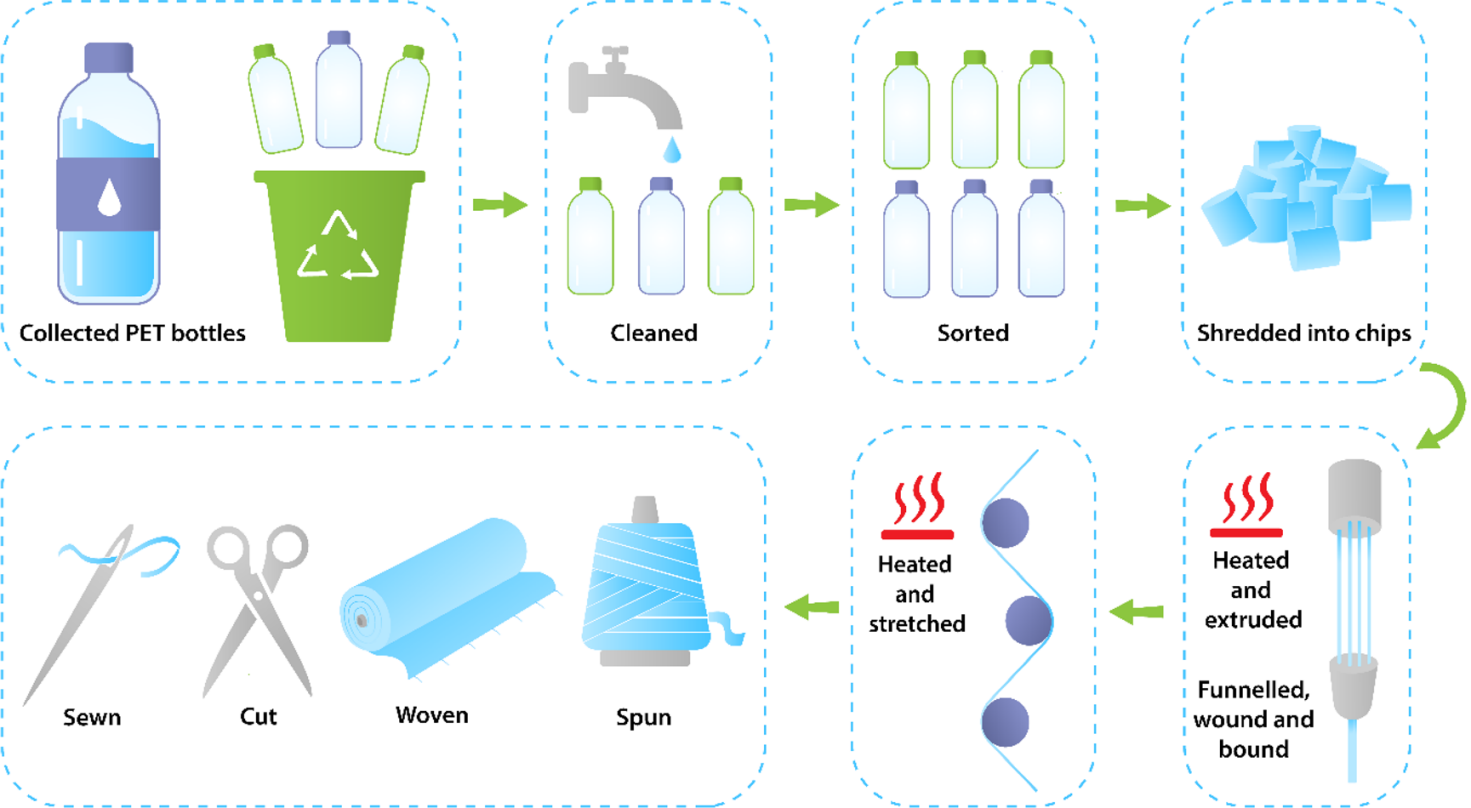
Process flow: from collection of PET bottles to recycling into garments.^64−67^

The growing concerns over environmental issues such as polymer waste, high energy wastage, greenhouse gas emission, marine plastic pollution, and other problems associated with plastic production^55^ have focused interest in the recycling of PET not only in the plastics industry but also within the healthcare wearable electronic devices sector.^56^ PET bottles can be recycled, with 94% of UK city councils now collecting such bottles either from the domestic home or at recycling centers.^57^ Nowadays, the importance of recycling PET bottles through both mechanical and chemical processes has been recognized,^58^ as well as the need to establish circular usage and improved end-of-life disposal.^59^ PET, a semicrystalline thermoplastic, is the most widely used polymer for industrial applications such as textile fibers and food packaging because of its superior mechanical, chemical and barrier properties, combined with good processability,^60^ low cost, and recyclability.^61^ Indeed, postconsumer PET bottles have become a popular choice for recycling into useful products such as textile fibers due to their chemical stability, mechanical strength, and fluid resistance. In addition, the thermoplasticity of PET provides a high degree of malleability and ductility at relatively low temperatures to allow easy molding and shaping. Together with its high biocompatibility, recycled PET (rPET) should be attractive and advantageous for use in wearable devices. rPET, which is mainly made by mechanical^62^ or chemical^63^ recycling of postconsumer PET bottles,^64−67^ is a sustainable choice for manufacturing textile fibers^68^ and meets the criteria for recyclable sustainable fibers either fully or partially, as illustrated in Figure 2.

Many textile businesses have already started to move away from virgin PET to rPET-based polyester fabrics with the benefits of less environmental impact. In fact, reports have confirmed that rPET consumes ∼70% less energy, and the CO_2_ emissions are ∼75% lower when compared to that of virgin polyester production.^69,70^ However, like virgin polyester, rPET-based textiles are non-biodegradable. Additionally, rPET polymers often suffer from poor mechanical performance due to the mixing of different grade polymers and subsequent thermal aging, resulting in phase separation, which adversely affects the material properties.^71,72^ Therefore, there remains a need for improving the melt-processability of rPET to adequately facilitate recycling. The performance modification techniques for recycled thermoplastic polymers have extensively been reviewed^71^ and include the use of additives such as compatibilizers, coupling agents, and impact modifiers. Among them, carbon nanotubes (CNTs) and graphene are of particular interest for wearable e-textile applications due to their capability in forming electronic networks coupled with their extraordinary mechanical and thermal properties. Such materials have already been used as both reinforcing agents and compatibilizers in polymer blends. Previously, we demonstrated significant improvements in mechanical and electrical properties of textiles fibers, yarns, and fabrics by adding graphene-based materials such as graphene oxide (GO), reduced graphene oxide (rGO), and graphene-based flakes,^9,73−75^ which can be extended to recycled polymers such as rPET to enhance its performance and functionality.

Further, in a recent study, a textile-based electrode was developed for a wearable supercapacitor by incorporating rGO flakes and polypyrrole (PPy) particles onto PET fabric, thus demonstrating its potential as a suitable substrate for wearable e-textiles applications.^76^ In another study,^77,78^ flexible MgO barrier magnetic tunnel junctions exhibited improved performance on PET substrate. The benefit of PET is its ultraflexibility and can be shaped into tubular form without compromising the performance of embedded electronics potentially,^77,79^ which could facilitate for the development of various lightweight and flexible wearable devices.^80^ The enormous potential for PET materials incorporating active conductive materials has been a focus for wearable electronics, e-textiles and e-skin in the application of strain sensors, smart clothes, health monitoring sensors, and human-machine interaction systems (Table 1). By effectively integrating the recyclability of PET with its compatibility with electronic components, PET offers many opportunities as the base substrate for wearable electronic devices.

**Table 1:** Summary of PET and PLA Polymers Used and Their Performance for Wearable Electronics

type of fiber/yarn/textile substrate	type of conductor materials	integration in textiles fabrication method	performance criteria	smart wearable electronic functionality	ref
PET	Graphene Nanoplatelets (GNPs)	spray coating	gauge factor (GF):150	piezoresistive - strain sensor	(113)
PET substrate	conductive Ag and CNT ink	flexographic printing		printed wearable sensors	(114)
PET substrate	active material: gold nanoparticles	inkjet		position: facemask	(115)
				printed respiratory rate sensors: resistive humidity	
PET	silver	screen		position: wrist	(116)
				printed temperature sensors: resistive	
PET	CNTs and PEDOT: PSS	screen	temperature sensitivity: 0.13%/°C	position: chest-resistive	(117,118)
PET	polymer paste	screen	capacitive: 9 kHz/°C	capacitive sensor	(119)
PET	CNTs	screen		position: chest printed heart rate and SpO_2_	(117,118)
				sensors: ECG/3 electrodes	
PEN, PET, ITO-coated glass	organic materials	blade-coating spin-coating	error: HR: 1%	position: finger	(120)
				HR/SpO_2_	
PET substrate	GNPs		up to 65% extensibility with a gauge factor of 62.5	piezoresistive strain sensor, smart clothes, touch-based flexible screens, flexible sensors, electronic skin, health monitoring, human/machine interaction system	(121)
			High sensitivity, long-term stability, reproducibility and durability.		
polyester (PET) fibers	scaffold PU fiber	coating with GO, winding	showing GF of 10 within 1% strain and high stretchability of 280%	strain sensor	(122)
PET yarns	coated with intrinsically conductive polymers PEDOT encapsulated in PMMA	fabrication: in situ polymerization	interconnectivity between the PET/PEDOT monofilaments enhanced on straining, decreasing resistance	fiber-based strain sensors	(123,124)
			resistance: ∼600 Ω/cm		
			GF: –0.76 (20% strain),0.665 (50% strain),–0.244 (70% strain)		
			durability - withstood 1000 cycles with stable GF		
PU core/PET wrapper fiber yarns and grapheme oxide (GO)	coated with graphene	dip-coating, when strained, GO/PET winding fibers separated, contact area decreased, resistance increased	conductivity: ∼0.15 S/m	health and motion monitoring	(122,124)
			GF: 10 (1% strain), 3.7 (50% strain)	detecting full range of human activities	
			response unchanged after 1000 cycles	fiber-based strain sensors	
			detection limit 0.2% strain		
			response time: <100 ms		
			reproducible signal up to 1310,000 cycles		
PU core/PET wrapper fiber yarns	coated with metals AgNWs	dip-coating, when strained, AgNW layer cracked, resistance increased	GF: ∼3.2(30% strain)	e-skin: fiber-based strain sensors	(124,125)
			sensed up to 50% strain		
			strain detection limit 1%		
PU core/PET wrapper fiberyarns and	coated with metals: AgNWs, silicone	dip-coating, when load applied to fiber crossover point, dielectric thickness decreased, resulting in change in capacitance	*S*: 0.096 kPa^–1^ (<0.1 kPa) and 1.1 MPa^–1^(0.1–10 kPa)	e-skin: fiber-based pressure sensors application	(124,125)
			durability: 10,000 cycles		
			extensibility: up to 30–40%		
			dynamic sensing range up to 50 kPa		
			detection limit of 1.5 Pa		
			response time ∼32 ms		
PET nonwoven fabric substrate	reduced graphene oxide (RGO)	suction filtration	stability (cycle):150 (10% strain)	strain sensor:monitor human wrist movements	(126,127)
			electrothermal property (about 50 °C under a voltage of 6 V)		
weft-knit polyester fabric	RGO	dip-coating	GF : 1.7 (<15% strain, *x*-direction), 26 (<8% strain, *y*-direction)	stain sensor: monitor physiological activities of a human body	(127,128)
			sensing range (%): up to 50		
			stability cycle: 500 (7.5% strain, *x*-direction), 500 (5% strain, *y*-direction)		
polyester knitted elastic band	RGO	dip-coating	GF: 34(0–20% strain), 5 (20–50% strain)	strain sensor: monitoring of large- and small-scale human body movements	(127,129)
			sensing range (%): 0.2–50		
			stability (cycle): >6000(30% strain)		
double-covered yarn (PU core fiber and polyester fibers)	RGO	dip-coating	GF:10 (<1% strain), 3.7 (<50% strain)	strain sensor: monitoring of a wide variety of human activities and complex robot movements	(122,127)
			sensing range (%): up to 100		
			stability (cycle): 10,000 (30 and 50% strain)		
PET or polyester fiber	graphene films		providing reliable electroanalytical performance with high flexibility, biocompatibility, wearability, and high detection sensitivity even under different motion states	flexible electrode for ECG monitoring	(130)
PET-based nonwoven material	graphite fillers	wet-laid method	excellent electrical and thermal conductivities, suitable flexibility	electrical devices and the potential for use in wearable devices	(131)
PE/PET nonwoven fabrics	graphene layer, silver nanoparticles (AgNPs)	two-step method: dipping process, magnetron sputtering	KH-560 treatment improves the interfacial adhesion between graphene and the PE/PET continuing to the enhanced the durability of the conductive composite fabrics.	wearable electronics applications	(132)
			graphene and AgNPs gave GPP and AGPP excellent thermal stability		
PET fiber surfaces	aqueous mixture of graphene oxide and AgNO_3_	dip-coating and subsequent reduction with hydrazine, thermal annealing at relatively low temperatures (<200 °C)	good durability of rGO coating on PET via a series of wash fastness and bending tests	improve the conductivity and durability of modified PET fabrics	(133)
SiO_2_/Si wafer/Cotton fabric/PET fabric	chemical vapor deposition (CVD)-grown graphene	colamination, wet transfer method	show smooth and homogeneous morphology, The sheet resistances of graphene on PET showed values comparable to those of graphene on the wafer	graphene e-fabric	(134)
				wearable chemical sensor showed a sensitivity up to 53% for nerve chemical warfare agents (GD)	
PET substrates	graphene type: GO films		motion detection by wearable sensors based on graphene materials	sensor types: piezoresistive	(135,136)
			motion types: elbow bending		
			demonstrate stable mechanical and electrical performances		
PET Substrates	CVD-graphene		finger touching	capacitive	(136,137)
PET substrates	bilayer graphene		touch movements	piezoresistive	(136,138)
three-arm stereocomplex PLA (tascPLA) substrate/dielectric layers	organic field-effect transistors (OFET)	spin-coating or dip-coating	transparency, flexibility, thermal stability, high-temperature sensitivity, degradability, and biocompatibility	biomaterial-based OFET, skin-like temperature sensor array	(139)
PP, PLA, PP/PLA composite	polydopamine (PDA)-treated and poly(3,4-ethylenedioxythiophene):poly(styrenesulfonate)	dip-coating, wet-spinning, scalable fabrication	strain sensing to monitor the tiny movement of human motion for public safety, healthcare, artificial muscles, military, space exploration, stretchable displays, sports, and consumer fitness	biodegradable, highly stretchable and wearable conductive yarn	(112)

Recently, bio-based and biodegradable polymers have emerged as latest classes of sustainable and natural^44^ materials (Figure 1b) as alternatives to the current fossil fuel-based fibers.^45^ The so-called “biopolymers” can degrade at a much faster rate than other conventional plastics.^83^ In addition, biodegradable bio-based polymers decompose into carbon dioxide, water, and other naturally occurring materials, leaving no residual toxins. Therefore, biopolymers could potentially meet growing demands for environmentally sustainable materials^48^ (Figure 1a) for fiber production.^84^ Furthermore, such bio-based and biodegradable polymers can address the problems of resource depletion^85−87^ and the environmental impacts of a fast-growing economy.^85,88,89^

**Figure 3:**
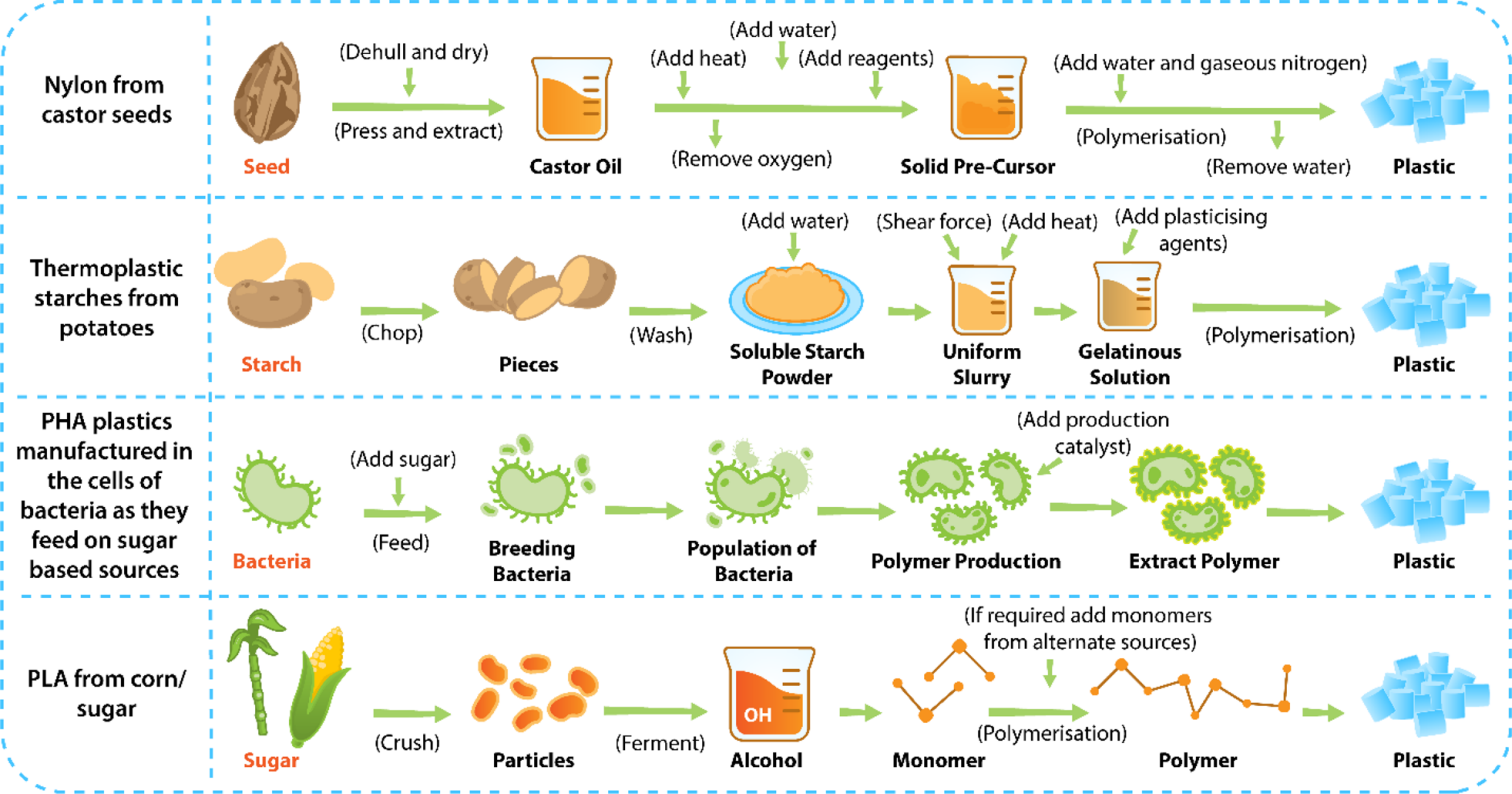
Different production routes for bio-based and biodegradable polymers.^81,82^

Previously, bio-based polymers have been mainly derived from agricultural feedstocks such as corn, potatoes, and other carbohydrate feedstocks.^90,91^ In addition, there have been extensive technological developments in renewable resource-based biopolymers, and their commercial applications have been studied.^92,93^ There are several methods that have been advanced over the years to derive bio-based and biodegradable polymers.^43^ The most commonly used production routes, Figure 3, includes^81,342^ (a) chemical conversion of natural raw materials into reactive monomers followed by polymerization, such as the process of obtaining nylon (amino acids) from castor seeds; (b) direct extraction of biopolymers such as starch and cellulose, followed by thermopressing/molding to derive thermoplastic starch polymers (TSPs); (c) further functionalization of extracted biopolymers by processes such as acetylation, carboxymethylation, and phosphorylation to make calcium alginate (CA), carboxymethyl cellulose (CMC), and cellulose diphenyl phosphate, respectively, which are then polymerized further;^94^ (d) hydrolysis of extracted biopolymers to sugars for the bacterial synthesis of polyesters, such as polyhydroxyalkanoates (PHA); (e) fermentation of the aforementioned biopolymer-derived sugars to lactic acids followed by their direct polycondensation or ring-opening condensation to PLA.^95^

**Figure 4:**
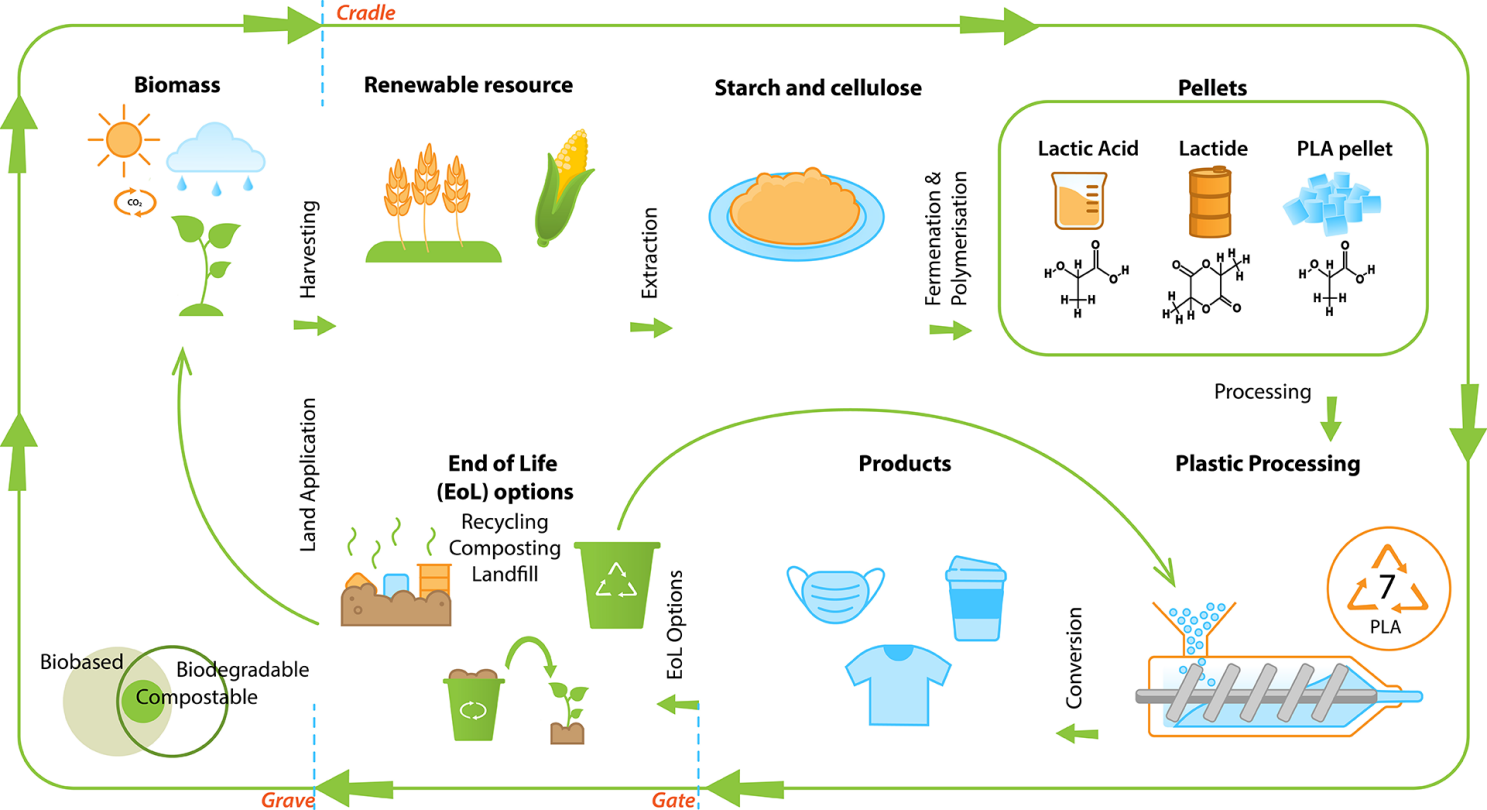
Life cycle of PLA from cradle to cradle.

According to standards developed by the Biodegradable Products Institute,^96^ PLA is an excellent biodegradable material as well as a green bio-based polymer. However, PLA will only degrade into carbon dioxide and water within a controlled composting environment,^97^ making it more sustainable than other types of plastics derived from fossil fuels and any other bio-based and biodegradable polymers. PLA is one of the most prevalent pervasive bio-based polymers for scores of usages, with diverse end-of-life (EoL) options (Figure 4), including recycling, landfilling, and industrial composting.^98^ In spite of this attractive performance and process flexibility, PLA does have some drawbacks. PLA are decomposed or sent to landfill after their useful life, due to an inadequate PLA recycling facility. PLA in this form is often considered “unidentifiable” and serves as a contaminant for the recycling of other plastics. Therefore, nonrecycled PLA products must be processed in an industrial compost environment to fully utilize its biodegradability, otherwise it may take many hundreds of years to decompose^99^ and may pollute the environment.

Nonetheless, by its nature, PLA possesses many desirable material properties and processing benefits.^100^ Thermoplastic PLA has a lower melting point than other petrochemical plastics and requires less energy for conversion, making it compatible with blow-molding and melt-spinning. PLA-based fabrics can also be pigment printed and thermally cured at different conditions.^101^ In addition, such fabrics can be inkjet-printed with UV-curable inks with good color strength and fastness properties.^102^ The outstanding processability of PLA makes it a preferred material for 3D printing. Also, while there are some concerns over the adsorption of viruses and molds to the surface^103^ of conventional biodegradable plastics,^104^ PLA shows excellent antibacterial,^105,106^ and antifungal properties. Furthermore, the PLA film also offers good air permeability,^107^ oxygen permeability^108,109^ and odor isolation.^110^ All these qualities promote PLA as a highly suitable candidate for use in wearable e-textiles. In a recent study, flexible and conductive membranes with antibacterial properties have been reported, showing potential for wearable movement sensing applications.^111^ The processability of PLA enables various form factors, such as PEDOT:PSS-coated conductive and stretchable PLA yarns could be used for detecting small movements in human motions.^112^

### Sustainable Electronic Materials

Wearable e-textiles incorporate electronic components and interconnections within their fibrous network^4^ to accomplish specialized tasks such as health monitoring. E-textiles have attracted considerable academic, military, and commercial interests because of their potential applications in defense uniforms, sportswear, and medical clothing.^140−142^ Much research has been carried out to integrate electronics into clothing for various applications including sensors,^122,143−145^ energy storage devices,^79,146^ transistors,^147^ heating textiles, electrostatic discharge clothing,^148^ physiological monitoring,^131^ photovoltaic devices,^149,150^ and many other critical applications. Importantly, for the end user, the e-textiles should offer the same properties as conventional textiles, such as washability, flexibility, low weight, and robustness, to be considered as fully “wearable.” These features depend on the properties of the electronic materials as well as the fibrous materials, which may be influenced by the post-treatment and integration techniques.^151^ Such smart e-textiles are manufactured either by printing or coating of electronic materials on the textiles surface or by incorporating such electronic materials within the fibers that construct the fabric. As a result, electronic materials and their applications in e-textiles are expected to drive the demand for promising textiles, fibers, fabrics, and processing technologies. However, even with their level of importance, there is little appreciation of how the use of electronic/conductive materials, such as gold, silver and copper, can involve large energy consumption, carbon emissions, overconsumption of natural resources and environmental pollution. Therefore, innovations in sustainable technologies are required in this sector, such as developing appropriate recycling strategies, utilizing biomaterials where possible, and discovering innovative materials. This review focuses on different electronic materials, including conductors, semiconductors, and dielectrics, identifying the challenges with their design, materials selection, and environmental impacts that must be overcome to achieve sustainability.

### Conductors

Conductors are the most important component in e-textiles, operating the electronics and facilitating the added functionalities. Metal inks based on silver (Ag),^152^ copper (Cu),^153^ or gold (Au)^154^ are right away the uttermost frequently used materials considering their high electrical conductivity (σ), commonly ∼10^5^ S/m.^155^ However, such inks are neither cheap^156^ nor environmentally friendly.^156^ They are not biocompatible^157^ and frequently need higher sintering temperatures,^158^ which limited the choice of textiles substrate. Therefore, there remains a need for an inexpensive and eco-friendly conductive materials that can be sintered at lower temperature for establishing sustainable wearable e-textiles. In the section that follows, we review the characteristics of such potentially sustainable metal-based, conductive polymer-based and carbon-based materials that are electrically conductive as well as some of their prospective uses in biodegradable wearable electronics.

#### Metal-Based Conductors

Electrode materials transport electrical charge carriers across electronic or sensing devices, as well as the external circuit. Conductors with immense electrical conductivities (>10^–1^ S/cm) are required to underpin a high-performance electronic device. It is becoming extremely crucial for “green” electronics formation to use biodegradable “metallic” component materials. To maximize the performance and reliability of the integrated circuit for wearable electronics applications, an optimum metal would have high carrier mobility, low resistance, significant thermal conductivity, outstanding mechanical properties, as well as corrosion resistance. Additionally, the changes in electrical properties as the metal degrades are the most important parameters that need to be considered for biodegradable metals. A previous study reported substantial standards for the use of metals in order to improve the performance of “green” electronics, which “dissolve” once their service life has been reached. It was shown that the electrical dissolution rate (EDR) of electrodes based on magnesium (Mg), Mg alloy, and zinc (Zn) boosted into salt solutions, or even the EDRs risen to body temperature (between RT and 37 °C), ceasing to increase within 8 h upon contact with a transistor.^159^ Conductive metals and microelements, including Mg, molybdenum (Mo), tungsten (W), iron (Fe), Zn, and their alloys,^160^ are a fascinating category of materials for short-term healthcare applications.^161^ Such materials have appealing electrical and mechanical properties, as well as degradability in a controlled environment.^162^ In addition, such materials can dissolve at different rates across both deionized (DI) water and simulation model biological fluids (Hanks’ solution pH 5–8),^163−165^ where metals move as electron donors and water as electron receivers.

One of the biggest challenges in developing wearable electronic devices is the requirement for flexibility, lightweight, thinness, safe and portable energy storage devices. To this end, we may draw inspiration from zinc ion batteries, which have demonstrated excellent potential for wearable electronics applications because of their safety, economical, and environmental friendliness.^166^ Zn dissolution rates for aqueous system and biofluids have been reported as 1.7 nm day^–1^ and 7.2 nm day^–1^, respectively. In addition, Mg has shown biodegradability and electrical conductivity as an electrode material for resistive switching memory devices based on chitosan,^167^ with both of these materials exhibiting rapid dissolution rates. In contrast, Mo and W are favored metals for degradable medical devices with transient lifetimes, such as physiological electrical signal sensing that may stand in need forthright connection amid biological tissues and metal parts. They have slower and more tunable degradation characteristics, around ∼10^–2^ nm day^–1^,^162,168,169^ making them better suited for controlled-length monitoring. Thin iron films show slow degradability, despite their rapid oxidation in biological conditions to convert into the iron oxides and hydroxides, which minimizes their solubility, The prolonged decomposition of these byproducts may restrict the use of Fe-based materials in certain biodegradable health monitoring applications, particularly oral or implantable techniques, which should vanish completely once their needs are fulfilled.^162^ In Table 2, the dissolution rate of inorganic conductive materials and their dissolution factors are summarized.

**Table 2:** Dissolution Products, Rate, and Factor for Metal-Based Conductive Materials

metallic conductor	dissolution product/solution	dissolution rate	dissolution factor	ref
Zn, Mg, Mg alloy	Zn(OH)_2_, Mg(OH)_2_	300 nm/day	room temperature, 37 °C, ceased within 8 h with MOSFET film	(157,159)
Mg	PBS solution		dissolution kinetic depends on the temperature and pH	(170−172)
			pH increases from 7.4 to 10, temp decreases from 37 to 25 °C	
			dissolution over 2 min to 12 h	
Mo, W	H_2_MoO_4,_ H_2_WO_4_,	∼10^–2^ nm/day	pH and temperature of the surrounding environment	(162,168,169)
Zn	Zn(OH)_2_	1.7 nm/day	pH and temperature of the surrounding environment	(162,168,169)
Zn	viofluid solution	7.2 nm/day	pH and temperature of the surrounding environment	(162,168,169)
Fe	Fe(OH)_2_		pH and temperature of the surrounding environment	

While Zn, Mg, Fe, W, and Mo have demonstrated excellent dissolution performance for many applications, including health monitoring devices, flexible electronics, energy storage, and electronic sensors, further studies will be needed to investigate their compatibility and interactions with sustainable textiles fibers for wearable e-textile applications. Additionally, suitable processes need to be established to either separate such materials from textiles or achieve complete biodegradation of both fibers and materials.

#### Conductive Polymer-Based Conductors

**Figure 5:**
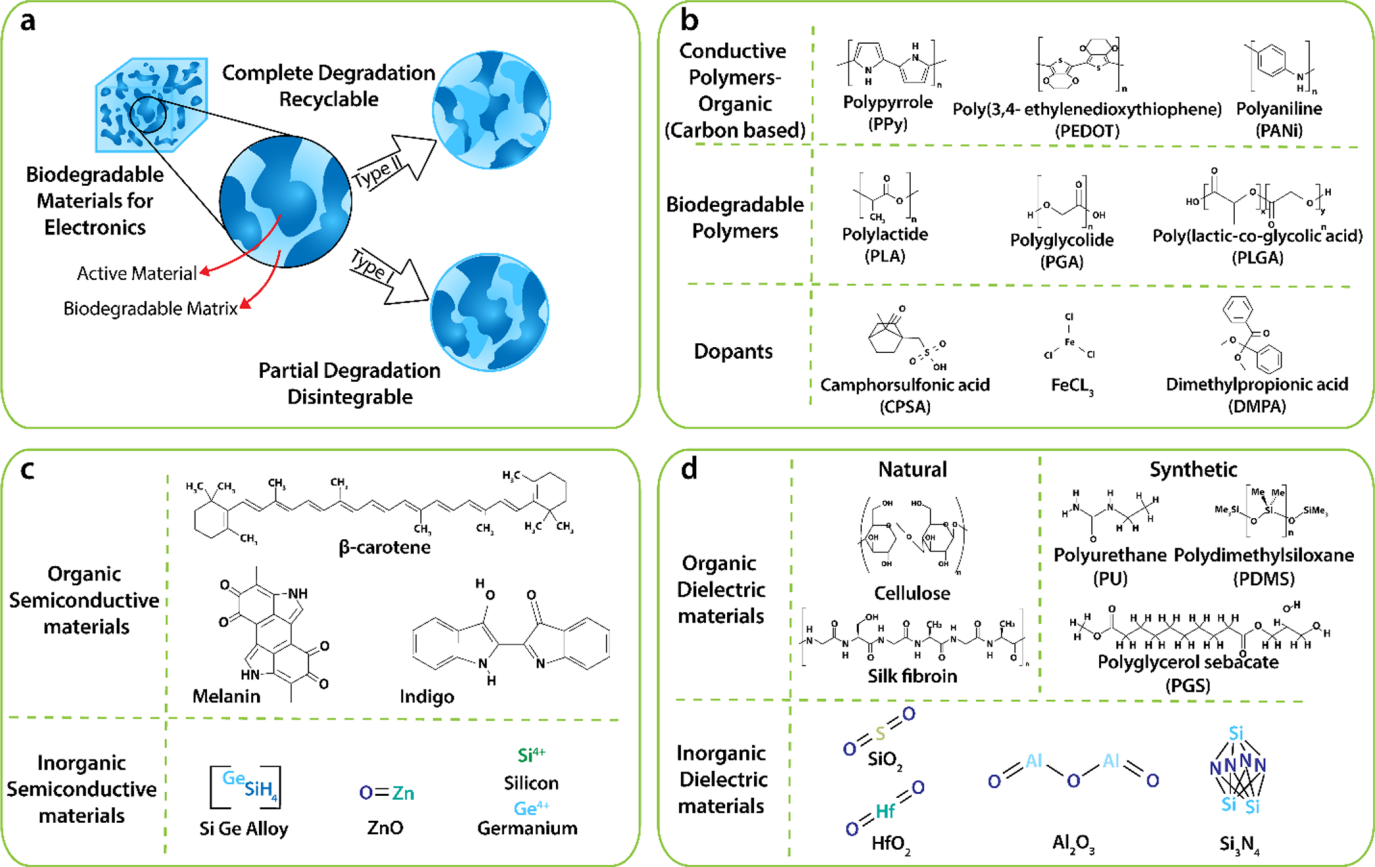
(a) Biodegradable materials with preferred electronic properties are made up of an active material (dark blue) dissolved within a biodegradable matrix (light blue), with biodegradable materials classified into type I and type II. Reprinted with permission from ref (185). Copyright 2018 American Chemical Society. (b) Chemical structures of biodegradable conductors. (c) Chemical structures of biodegradable semiconductor. (d) Chemical structures of dielectric fillers.

Conductive polymers (CP) are electrically conductive materials composed of a conjugated covalent backbone and functional groups with pseudocapacitive characteristics, together giving rise to conductive properties.^173^ The most common conductive polymers are polypyrrole (PPy), polyaniline (PANI), polythiophene (PT), and PT derivatives such as poly(3,4-ethylene dioxythiophene) (PEDOT), which have been used satisfactorily as electrode materials (Figure 5b).^174^ Such conductive polymers have mechanical flexibility greater than that of metal contacts, which makes them ideal for developing flexible and conformal electronics for health monitoring systems.^162^ Additionally, they have demonstrated excellent biocompatibility within biological systems.^175^ Furthermore, the properties of conductive polymers are beneficial in wearable devices focused on on-skin sensing.^176^ Textiles or films with conductive polymer coatings are suitable for use in various military and aviation applications, as the conductive polymer components have the combined properties of both metals and plastics. In studies,^177−180^ efficient *in-situ* polymerization of CP precursors such as aniline and pyrrole on the textile substrates has been achieved, resulting in electrically conductive cotton and polyester-based textiles. By combining inherently conductive polymers with more mainstream fibrous polymers, the characteristic mechanical and physical properties of the textile can be produced.^181,182^ Such polymeric combinations may better suit wearable technologies, due to their inherent mechanical stiffness, flexibility and adaptive structure with stretchable electronics.^3,183,184^

Although CPs are suitable for many electronics applications, conventional CPs are still nondegradable, and their sustainability needs to be improved for imparting reliable electrical conductivity while reducing comparable concentration of the nondegradable conjugated component.^185^ One method of dealing with the deterioration of these conductive polymers would be to construct a composite structure where the conductive polymers are embedded in a biodegradable insulating matrix. Under right set of circumstances, such matrix can be degraded, and the nondegradable components are biocompatible.^185,186^ This type of degradation is classified as type I, as illustrated in Figure 5a, with typical examples (Table 3) such as PANI, PPy, and PEDOT which have conductivities up to 4.6 × 10^3^ S/cm when doped with poly(styrenesulfonate) (PSS).^172,187^ The second approach is to incorporate flexible, biodegradable and conductive polymers and nonconjugated division with the copolymer backbone format. The obtained small and conjugated components after breakdown are nontoxic, and they could further be degraded or eliminated by the atmosphere or the body’s immune function. Such degradation is labeled as type II,^185,186^ as illustrated in Figure 5a, and biodegradable polyurethane segments are typically incorporated into the copolymer backbone to achieve type II (a few examples are given in Table 3) degradation.^186^ These biodegradable conductive polymers have recently attracted considerable attention as an evolving class of futuristic polymeric biomaterials that offer electrical conductivity, biocompatibility, and biodegradability. Conductive polymers such as PANI, PPy, PT, and biodegradable polymers such as poly(d,l-lactic acid) (PDLLA) and PCL can be covalently bound together, enabling the ability to “tailor and engineer” the performance and sustainability properties of the final polymeric material. However, an ongoing challenge for organic-based conductive polymers is that the material’s longevity is relatively poor, specifically the inadequate cycling performance of conductive polymers in applications such as supercapacitor electrodes^173,188^ in wearable electronics. To overcome this technical deficiency, incorporating carbon-based materials into conductive polymers is a promising approach to enhancing their overall electrochemical performance by integrating the optimal individual properties of both components leading to better utilization and sustainability.

**Table 3:** Types of Conductive Polymer Degradation^a^

conductive polymer	material system	dopant	conductivity	applications	ref
type I: conductive blends	PANI gelatin nanofibers	camphorsulfonic acid (CPSA)	2.1 × 10^–2^ S/cm	scaffold for tissue engineering	(185,196)
	PANI electrospun with PLCL	CPSA	1.4 × 10^–2^ S/cm	control of cell adhesion	(185,197,198)
	PPy with PCLF (polycaprolactone fumarate)	anionic dopants: napthalene-2-sulfonic acid sodium salt and dodecyl benzenesulfonic acid sodium salt	6 × 10^–3^ S/cm	nerve regeneration	(185,199,200)
	PEDOT particles in PLLA	hyaluronic acid	4.7 × 10^–3^ S/cm	biomedical application	(185,201,202)
	PPy nanoparticles in PDLLA	oxidation with FeCl_3_	1 × 10^–3^ S/cm	biosensors, drug-delivery systems, biomedical applications, as well as tissue engineering	(185,203,204)
	PPy-coated PLGA fibers	oxidation with FeCl_3_	*R*_s_ = 4.7 × 10^5^ Ω/sq	neuronal tissue scaffolds	(185,205)
type II: conjugation breaking	PANI grafted to gelatin	CPSA	4.5 × 10^–4^ S/cm	tissue engineering	(185,206,207)
	PPy-thiophene-PPy with aliphatic linkers	iodine	10^–4^ S/cm	electrochemical energy storage, as well as optoelectronics	(185,208,209)
	hyperbranched: AP and PCL	hydrochloric acid (HCl)	2.4 × 10^–5^ S/cm	tissue repair and bioelectronics.	(185,210)
	aniline trimer with polycaprolactone	dimethylpropionic acid (DMPA) (incorporated into backbone)	1.2 × 10^–5^ S/cm (dry)	skeletal muscle tissue engineering	(185,211)
			4.7 × 10^–3^ S/cm (in PBS)		
	aniline tetramer grafted to poly(ester amide)	CPSA	8.0 × 10^–6^ S/cm	vascular tissue engineering	(185,212−214)
	aniline pentamers (AP) with PLA triblock copolymers	CPSA	5 × 10^–6^ S/cm	tissue engineering	(185,215−217)
	quaterthiophene and alkyl chains joined by ester bonds	FeCl_3_ and Fe (ClO_4)3_			(185,218)

a Composites of nondegradable conductive polymers and electrically insulating degradable polymers make up type I conductors. Type II conductors are created by cleavable linkages, connecting conductive oligomers with degradable polymer segments. Dopants must achieve workable conductivity values in both case scenarios, and they need to be biocompatible.

#### Carbon-Based Conductors

Carbon-based materials such as single wall/multiwall CNTs and graphene have been reported as good conductors for wearable electronics applications.^172^ Due to their noticeable features, such as nanoscale diameters, outstanding electrochemical functions, electrical conductivity, favorable physical properties and biocompatibility, CNTs and graphene can be used for fabricating electrochemical devices.^189^ Carbon-based conductive materials (CNTs, carbon fibers, graphene, reduced graphene oxide, and pyrolytic carbon particles) are also thermally stable and have electrical conductivities (of CNTs and graphene) greater than those of the conductive polymers.^190−193^ Furthermore, virtually any known form of applicable conductive carbons can be formed from renewables.^193^ Therefore, the use of carbon-based conductive materials is considered to the “greener” option than metals. However, the electrical conductivity of carbon-based materials is not as significant as metals, even though CNTs are an example of the most promising materials for wearable electronics offering metallic-like and superconductive electron transport^194^ and can be modulated under several forms of stimulation.^195^ This functionality can help monitor various health parameters such as temperature, heart rate, and glucose levels in the blood. In addition, CNT-based e-textiles may help to record electrocardiography (ECG), electroencephalography (EEG), and electromyography (EMG) signals.

In addition to building on the initial CNTs studies, graphene inks have also been assessed within electronic components and devices integrated into smart fabric textiles. This material changes the wearable electronics textile framework with its excellent physical properties for use in electronics, sensing, catalysis, photonics, and energy storage.^219^ Graphene’s atomic thickness (0.345 nm) and outstanding electrical and mechanical properties^220^ also offer further significant benefits, allowing deposition of exceedingly thin, soft, and conductive film on surfaces of textile fibers through inkjet printing. The strong adhesion of graphene to textile substrates, combined with the inherent environmental compatibility, makes graphene-enhanced electronics attractive for wearable applications. Moreover, incorporating carbon-based fillers further increases the host material’s performances^221^ with less material needed to deliver the same performance, thus improving sustainability. Hence, the carbon footprint required to manufacture and integrate the material into its intended application will be decreased. The use of such conductive materials in this framework can improve the sustainability of the recycled or biosynthetic polymer also by creating a more durable fiber, so fewer plastics will need to enter the landfill and, in turn, the oceans and local ecosystems.

### Semiconductors

Semiconductors are essential to the switching mechanism of organic transistors,^222^ and these are necessary for complicated electronic circuitry. They are usually distinguished by their charge carrier mobility (*μ*_*i*_), that reflects how rapidly a free charge can keep moving through the substances when dragged by an electric field. Mobility and conductivity (σ) are related by the following equation:
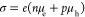
where *n* is the concentration of electrons with mobility μ_e_ and *p* is the concentration of holes with mobility μ_h_. Mobility is normally expressed in cm^2^/V·s and can be calculated directly from working devices like thin-film transistors. Regular semiconductive polymers are PT (e.g., poly(3-hexylthiophene), P3HT) and donor–acceptor copolymers developed originally for organic photovoltaics (e.g., diketopyrrolopyrroles, DPP).^223,224^

In some cases, a semiconductor-containing device can contain large quantities of hazardous elements such as mercury, chromium, arsenic, and lead (Pb), which can release toxic elements into soils and waterways when e-textile derived waste is thrown away in landfills. Additionally, the incineration of plastics used for semiconductors can lead to the discharge of volatile compounds like dioxin derivatives, polychlorinated dibenzofuran and polychlorinated biphenyls, that are classified as group 1 carcinogens.^227,228^ Therefore, to reduce the intensity and influence of waste electronics, there is a significant focus on sustainable semiconductors which contain more environmentally friendly materials.

Developed in a previous study,^229^ a *p*-phenylenediamine (PPD) polymer-based semiconductor degraded when exposed to a weak acid. The substrate was made of transparent cellulose, and the electronic components were comprised of iron rather than gold, because it is less toxic to humans and more eco-friendly. Skin-like electronics have a wide range of potential applications, including skin patches that can monitor glucose levels, blood pressure, and other vital signs. It can also act as a wearable device and can interact with ecologic detection systems that could be utilized across a vast wooded areas, sending back data on the forest “health” during the biodegradation.

For many applications, the total breakdown of polymers into their monomeric building blocks is redundant, and device disintegration is adequate to prevent obstructive and pricey recovery processes. Type I^185^ materials are those that exhibit transient behavior, as illustrated in Figure 5a and Table 1. Like conductive polymers, blending has been used to produce semiconductors which display type I degradation. Furthermore, the notion of blending has been investigated in order to generate flexible “green” electronics, in which two conductive polymers (CPs) are blended together to overcome the flaws of the individual materials.^230^ For type II biodegradable semiconductors, the innovative adoption of reversible imine bonds as conjugated linkages between DPP and p-phenylenediamine recently has been reported.^185^ Poly(3-thiophene methyl acetate) (P3TMA), a derivative of P3HT with carboxylate substituents, was selected for blending with poly(tetramethylene succinate), PLA, poly(ester urea), and thermoplastic polyurethane (TPU) to enhance miscibility with more polar, biodegradable matrixes.^185,231^ Therefore, we can expect fully degradable semiconductors, which signifies a good potential step toward the development of multifunctional materials for wearable electronic devices that can resolve earlier unconquerable obstacles and develop fit for purpose inventions.

#### Organic Materials

**Figure 6:**
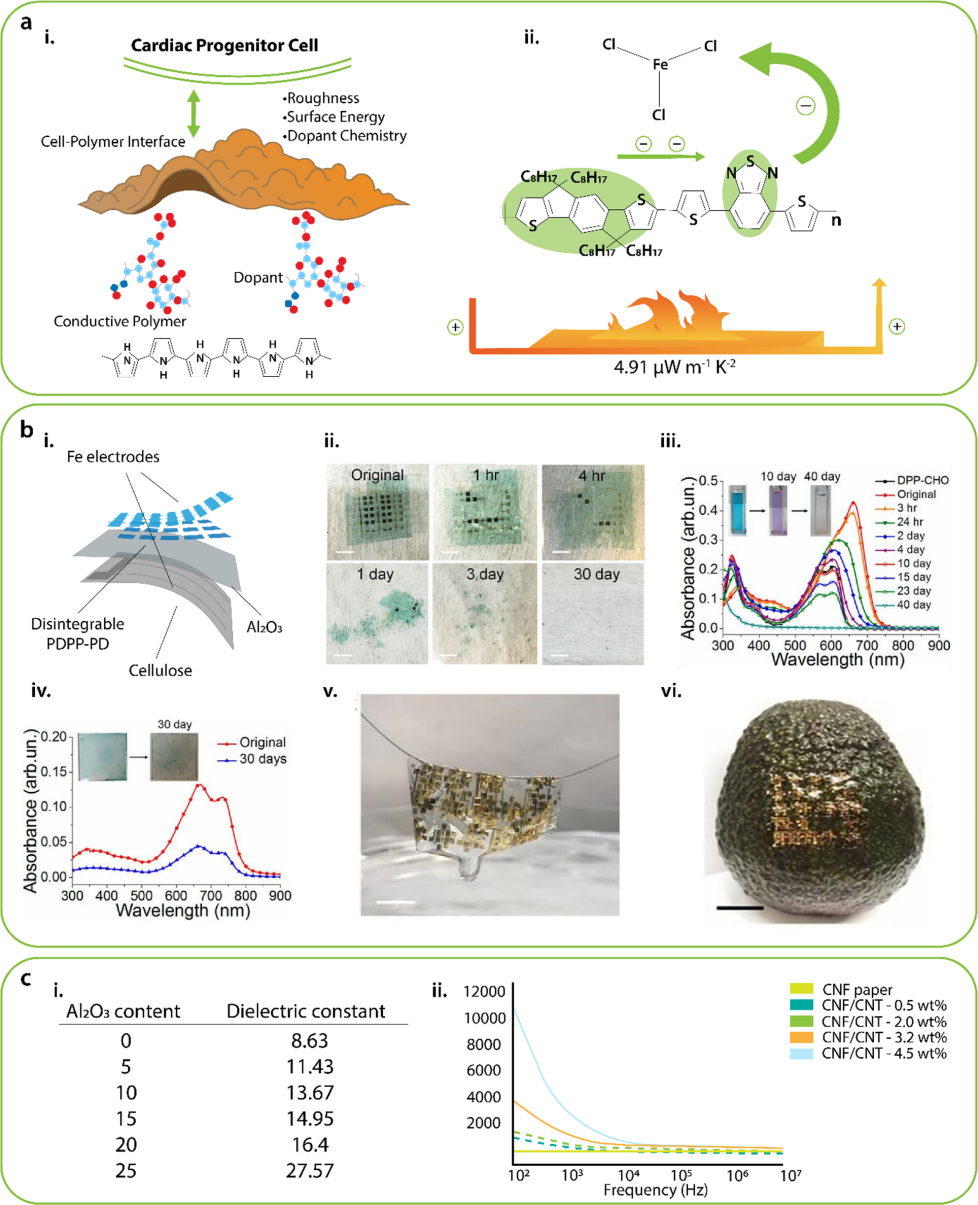
(a) Conductive polymer doping for degradation: (i) cardiac progenitor cell synergy with the conductive polymer (PPy) surface is critical for optimizing cardiac tissue regeneration.^225^ (ii) Conductive polymer (PANI) doping with FeCl_3_.^226^ (b) Disintegration, degradability, and biocompatibility of PDPP-PD: (i) Illustration depicting the materials and device structure used in total disintegration electronics with iron electrodes. (ii) Images of a device disintegrating at different phases. (iii) Absorption spectrum shifts during the decomposition of PDPP-PD. (Inset) Images of polymer solution change in color after 10 and 40 days of decomposition. (iv) Absorption spectrum of a polymer film before and after decomposition in a pH 4.6 buffer solution changes. Images of the film before and after decomposition (inset). (v) Strand of human hair picked up disintegrable pseudo-CMOS circuits based on PDPP-PD. (vi) Device was positioned on the rough surface of an avocado.^229^ (c) Dielectric constant of degradable composites can be enhanced by combination of high-κ additives such as (i) Al_2_O_3_ and (ii) CNTs. Reprinted with permission from ref (185). Copyright 2018 American Chemical Society.

The development of organic-based semiconductor materials as a replacement for traditional inorganic materials is a recent research focus. Their beneficial characteristic features include solution processability, superior mechanical flexibility, simplicity of structural modification, low-temperature fabrication method, relatively low charge, and the ability to produce on a large scale.^186,232^ Researchers have attempted to identify naturally occurring conjugated materials for use in the fabrication of semiconductors for electronic devices. Furan has shown some promise as a “building block” because of derivatives can indeed be made from natural sources,^233^ and the establishment of furan-based semiconductive materials may represent a major leap toward green electronics. Oligofurans have widely been reviewed (with a mobility of 10^–2^ cm^2^/V.s) as active semiconductor materials in OFET devices, and furan substructures have extensively been accounted as constituents within the backbone of conjugated materials.^234^ Bao et al.^229^ also designed a DPP-based polymer (polymer diketopyrrolopyrrolephenylenediamine, PDPP-PD) that recommended imine functionality into the polymer backbone to escalation biodegradability. Following that, this device demonstrated excellent hole mobility (0.12 cm^2^/V·s) and extremely good biodegradability. The device was reasonably stable in DI water disintegrated within 30 days immersed into a pH 4.6 buffer solution (Figure 6b).

Melanin, a conjugated biopolymer widely found in nature, provides a hybrid electronic-protonic conductivity.^172,235,236^ The conductivity of melanin is strongly reliant on its hydration state, with a fully hydrated state having a conductivity of 10^–3^ S/cm and a dehydrated state having a conductivity of 10^–9^ S/cm to 10^–8^ S/cm. Temperature and physical framework are also factors that influence its conductivity.^185^ Melanin, indigo derivative, purpurin, alizarin, PDI (pyridinediimine), and NDI (naphthalenetetracarboxylic diimide) carry a great number of C=O functionality that aids in forming intramolecular and/or intermolecular H-bonding interactions. Regardless of the absence of intramolecular conjugation in some of these molecules, the H-bonded intermolecularly connected materials provide outstanding quality for organic electronic devices, denoting good prospects as biodegradable semiconductors in future green electronics. β-Carotene is another noteworthy natural conjugated polymer, which make the orange color for carrots and can be used as a nontoxic semiconductive layer in organic bioelectronics.^237^ It has been proclaimed as a p-type field-effect semiconductor with charge mobility of 4 × 10^–4^ cm^2^/ V·s.^185^ Solution-processed p-type β-carotene was employed satisfactorily as an active layer for OFETs and organic solar cells (OSCs).^238^ As a result, much further exploration into the use of organic semiconductor materials is necessary to accomplish fully organic bioelectronics with enhanced electrical performances.

#### Inorganic Materials

Along with their own outstanding conductivity, mainstream semiconductors are primarily based on metal oxides and Si derivatives, such as various forms of Si and Si–germanium (Ge) alloys. The resulting conventional integrated circuit from such materials may not disintegrate for many hundreds of years, making them non-biodegradable materials. Nevertheless, some studies have shown that nanomembranes or nanowires of Si or Si/Ge alloy can improve biodegradability to facilitate the dissolution/decomposition process of the integrated circuits by fully decomposing within 10 days, without generating toxic byproducts.^186^ For instance, Rogers et al.^239^ investigated the transient form of Si by fabricating a set of functional devices, including resistors, field-effect transistors, photodetectors, and diodes, using a Si nanomembrane as the semiconductor.

Previous studies^241−247^ have shown that mono-silicon nanomembranes (Si NMs), amorphous silicon (a-Si), germanium (Ge), polycrystalline silicon (poly-Si), silicon–germanium alloy (SiGe), zinc oxide (ZnO), and amorphous indium–gallium–zinc oxide (a-IGZO) are soluble (Table 4) in biological aqueous medium. pH, temperatures, doping levels, concentrations, and types of ions and proteins in the solution are discovered each to have a major impact upon that dissolution rates of mono-Si NMs. pH levels and rising temperatures accelerate the dissolution rate of Si NMs, whereas a high doping level (>10^20^ cm^–3^) significantly slows the dissolution rate.^246,247^ Correspondingly, the dissolution rates of poly-Si, a-Si, Ge, and SiGe are largely shaped by pH, temperature, proteins, and ion type. Such materials, for instance, dissolve faster at physiological body temperature (37 °C) than at room temperature. For poly-Si, a-Si, and single-crystal Si, dissolution rates in bovine serum at a similar pH are 30–40 times higher than those in a phosphate-buffered solution at 37 °C.^245^ Si-NMs with coherent hydrolysis rates have found widespread application in biodegradable electronics devices such as diodes, photodetectors, and metal–oxide–semiconductor field-effect transistors (MOSFETs).^162,165,248^

**Table 4:** Dissolution Solutions, Dissolution Factors, Electron Mobility and Hole Mobility of Biodegradable Semiconductor Materials

biodegradable semiconductor materials		dissolved solutions	dissolution factor	electron mobility (cm^2^/V·s)	holes with mobility	ref
organic	DPP			up to 0.55	up to 0.34	(172,185)
	PDPP-PD	hydrolyzed with a catalytic amount of acid	30 days for disintegration in a buffer solution with pH 4.6		0.12	(186)
	anthraquinone derivatives			1.2 × 10^–2^		(185)
	β-carotene			4 × 10^–4^		(186)
	natural dye Indigo			10^–2^		(172,185,186)
inorganic	Si-NMs	biofluid and aqueous solution Si + 4H_2_O →Si(OH)_4_ + 2H_2_	time: 12 h,temp: 37 °C,pH: 6–14			(172,240)
			doping level of the Si, ionic concentration, hydrolysis rate of Si NMs is around 10 nm/day in groundwater			
	ZnO	produced Zn (OH)_2_ by hydrolysis, slightly soluble ZnO (s) + 2OH^–^ → ZnO_2_^2–^ + H_2_O				(172)
	Ge	produced H_2_GeO_3_ upon hydrolysis Ge + O_2_ (aq) + H_2_O →H_2_GeO_3_(aq)	dissolution rate of 3.16 nm/day, same physical condition as Si-NMs			(172)

Zinc oxide (ZnO) has indeed received a great deal of attention in published findings as an inorganic semiconductor owing to its large and direct band gap (3.37 eV) and high exciton binding energy (60 meV).^249^ ZnO, a piezoelectric material that is free of lead, is widely used in mechanical sensing and energy harvesting, with differing ZnO nanorod^250^ lengths both influencing and improving the nanogenerator energy-harvesting performance. Consequently, there is potential to efficiently convert from physical movement and the environmental elements to electronic signals using this dissolvable ZnO, which could drive wearable electronic textiles for sensing, activity recognition, or surveilling to raise the quality life.

### Dielectric Materials

Another essential building block for manufacturing wearable electronics are dielectric materials, which are necessary for facilitating capacitive functionalities. These insulating materials have the greatest impact on determining the overall properties of a component. Dielectric materials are characterized by the dielectric constant, *K*, defined as the ratio of the material’s electric permeability to free space’s electric permeability (i.e., a vacuum). The permittivity of a dielectric material comparable to that of free space is known as relative permittivity, generally denoted by ϵ_r_, the dielectric constant. The following equation connects absolute permittivity (ϵ_0_), relative permittivity or dielectric constant (ϵ_r_), and permittivity of a material (ϵ).
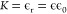
Whenever a voltage is applied to a capacitor, its dielectric constant determines how much energy it can store. Even if a dielectric material is exposed to an electric field, it becomes polarized, and polarization reduces the effective electric field. Because the permittivity of a material varies with frequency and temperature, the dielectric constant is administered at specific conditions, typically at low frequencies.^185,251^ Depending on the requirements, the target dielectric constant (*K*) can be high or low, usually aiming for low dielectric losses for minimal dissipation of electromagnetic energy and high breakdown voltages for stability.^185^

Incorporating high-*K* fillers into a degradable polymer matrix is a common strategy for developing biodegradable dielectrics. In addition to natural materials, such as cellulose and silk fibroin, poly(glycerol sebacate) (PGS), a synthetic biodegradable polymer, also has functional dielectric properties.^252^ Elastic materials (e.g., PGS) are advantageous for capacitive sensing as they can resist repeated and reversible deformation better than viscoelastic alternatives because textiles materials have a very low dielectric constant.^162^ For example, the dielectric constant for silk and polyester is 1.75 and 1.90, respectively, which are comparatively lower than those of SiO_2_, HfO_2_, and Al_2_O_3_ which are 3.9, 25.0, and 27.6, respectively. Textile materials generally have quite small dielectric constants because they are porous, as well as interstitial air raises the relative permittivity to be closer to unity (i.e., dielectric constant in air).^253^ A range of dielectric fillers are detailed in Table 5 with dielectric constant, frequency, and their potential applications as sustainable materials.

**Table 5:** Dielectric Constant Frequency and Applications of Dielectric Fillers^172,186,213^

dielectric fillers		dielectric constant (*K*)	frequency	incorporation/application
inorganic	SiO_2_	3.9		
	Si_3_N_4_	7		gate dielectric
				dissolved in 6 months in PBS at pH 12 and 37 °C
	Al_2_O_3_	27.57	50 Hz	cellulose acetate (Figure 6c)
	HfO_2_	25		
organic	cotton	17	60–1000 Hz	
	sugar-glucose	6.35	1 kHz	
	sugar-lactose	55	1 kHz	
	DNA and its precursors			gate dielectric, cationic surfactant—hexadecyltrimethylammonium chloride (CTMAC)
	PGS poly(glycerol sebacate			capacitive sensor
	CNTs	3198	1 kHz	CNFs (Figure 6c)

Currently, the potential for wearable e-textile has gained prominence in the healthcare sector, focused on applications in sensing, communication, health monitoring and simply following up with patients. This clothing-based communication platform^254^ requires the miniaturization of wireless devices for tracking and navigation, mobile computing and public safety. This communication is possible when the wearable antenna can transmit fast-changing signals across the textile transmission line based on the complex dielectric permittivity of the material or substrate. Incorporating sustainable dielectric materials such as SiO_2_ or Al_2_O_3_, which have high dielectric constant, into these wearable textiles can establish a latest sustainable opportunity for developing wearable antennae (Table 5). While the variable positioning of wearable antennas on the body due to human body movements, such as standing, sitting, walking, running and during sports, may affect the transmission of electrical signals, textiles infused with dielectric materials provides an opportunity to address this issue and improve the user experience.

### Sustainable Electronic Components

Electronic components are frequently encapsulated in discrete form with two or more running parallel leads or conductive pads. The functions of electronic components depend on the type and application of the circuits. Electronic components are projected to be soldered to a printed circuit board (PCB) to form an electronic circuit with a particular function.^255,256^ There are two categories of electronic components: passive and active. Passive electronic components include resistors, capacitors, diodes and inductors, all of which lack gain or directionality. In contrast, integrated circuits (IC), transistors, and logic gates are examples of active electronic members that have yield or directionality.^255^

Wearable electronics would not be feasible without electronic components, such as electrodes, connectors and interconnectors. For example, electrodes act as a connection between the body as well as the circuit when wearable e-textiles acquire electrical or biological signals. Even when electrical signal acquisition is not required, connectors and interconnectors are necessary to create a connection with both textiles and electronics. Textile circuits are usually built on textile substrates by printing, knitting, embroidering, or laminating. Embroidery of conductive threads into textile substrates to form electronic device is a common method for stitching patterns that designate component connection pads, circuit traces, or sensing surfaces employing computer-aided design (CAD) tools.^257^ There are many types of textile yarns available for creating electronic connections and circuit elements, including silver-coated yarns, gold, titanium, and stainless steel, and tin threads. Conductive patterns can also be made via inkjet printing of conductive inks.^258^ Textile circuits are generally constructed with a low-power rate and a large input impedance, which contrasts with the traditional requirement of low impedance for component interconnections. Hot press, a heat weldable electronic circuit to a textile substrate is another method for making textile circuits.^259^ Once connected to the textiles, the circuit can be soldered like a traditional PCB. Furthermore, conductive inks and polymers could be used to print flexible conduction lines.

Fixing the circuitry to textiles uses materials such as solder, protective coatings, plastics and paints. Solder is used in circuit board assembly to melt and form a solid metal joint between the pins of electronic components and the metal landing pads on the board. Soldering, on the other hand, can produce high concentrations (>85%) of lead^260^ as well as other toxic substances, attempting to make it hazardous to humans and the environment. To protect against these toxic materials, printed circuit boards can now be formed using green technology, which includes lead-free soldering.^241^ It not only provides a more sustainable and environmentally friendly option but also conforms to the restriction of hazardous substances (RoHS)^260^ legislation mandated for electronics sold in Europe.^261^

## Sustainable Manufacturing

The sustainability of wearable electronics depends on the sustainability of its individual component elements and the manufacturing process.^262^ In other words, the textile substrates, insulators (dielectrics), conductors, and semiconductors all need to be fabricated through sustainable techniques, as well as themselves be sustainable by being recyclable and/or biodegradable. Such resource-rich products need to be designed in such a way that they do not lose their inherent value at the end of their life cycles. Additionally, sustainable production requires that the energy, water and other natural resources used to manufacture such devices are optimally utilized as much as possible. Furthermore, the disassembly of various electronic components needs to be considered to fully recycle wearable electronics at the end of their useful life.^263^ The degree of integration of the numerous electronic components within the substrates has a direct impact on this goal. These electronic components can be embedded within the fibers or be removable. An assembly process that considers a lower level of integration with relatively low negative environmental consequences will be beneficial not just for reuse and recycling, but also for cleaning, washing, and updating rapidly changing technology, and can constructively involve the end-user throughout this process.^264^

There are several techniques available for incorporating electronic materials (conductors, semiconductors and dielectric) into traditional textiles, either in fiber, yarn, or fabric form.^265,266^ In the most simple sense, the process of incorporating conductive yarns into traditional textiles can be achieved by manually sewing the conductive yarns or mechanically embedding in the fabric through knitting, weaving, embroidery, or braiding.^4^ Nonconductive yarns can be coated with galvanic substances, metals, or metallic salts to make electrically conductive yarns from “pure” textile threads, enabling e-textile production.^4,267^

Over the past decade, it has also been evident that manufacturing techniques used to produce conventional textiles could be applied to produce e-textiles.^268^ For example, the fabrics used for protective medical clothing are typically knitted, woven, or nonwoven. Woven fabrics are generally stronger and more durable;^269,270^ they are less extensible, however, than knitted fabrics, which are porous but have relatively poor barrier properties. Knitting is the second most used fabric manufacturing method after weaving. Knitting is a faster and more cost-effective method of converting yarn into textiles than weaving. Furthermore, knitted fabrics typically offer improved comfort and a great choice in most sorts of apparel because of their ability for very high extensibility, up to 100%.^271^

Nonwoven fabric (NWF) is a low-cost textile material that can be engineered in higher value functionality in healthcare, protective clothing, filtration, and packaging. In a previous study,^272^ graphene enhanced NWF sensors have demonstrated that they can respond to a variety of human movements, with a clear differentiation of the activities’ levels of difficulty. They can also monitor small-scale movements such as pulse and respiration. Additionally, chemical vapor deposition, sputtering, electroless plating, and conductive polymer coating are also widely accepted textile coating processes.^273^ Several methods exist for printing conductive material on textile substrates, however they all use conductive inks containing metals such as copper (Cu), silver (Ag), and gold (Au).^274^ Many fabrication processes and materials make the wearable e-textiles field more promising by providing exemplary performance, such as lightweight, strong mechanical properties, high conductivity, wearability and biocompatibility. Even after these benefits, electrical and mechanical performance metrics such as stability, sensitivity, and long-term use are still lacking for realizing practical wearable applications. Accordingly, there is still considerable scope for improvement in wearable e-textile design through improved fabrication or manufacturing technology that can be cost-effective, environmentally friendly, and provide better performance. Among all the fabrication and manufacturing techniques, melt-spinning and digital inkjet printing are of particular interest due to inherent advantages such as less waste of materials, less water and energy-intensive processes, making such methods suitable for sustainable manufacturing of conductive e-textiles. Additionally, we note the potential of electrospinning, an emerging technology that has begun its transition from the laboratory-based incubation stage to commercial-scale development, for providing additional opportunities to integrate distinctive functionalities.

### Melt-Spinning

**Figure 7:**
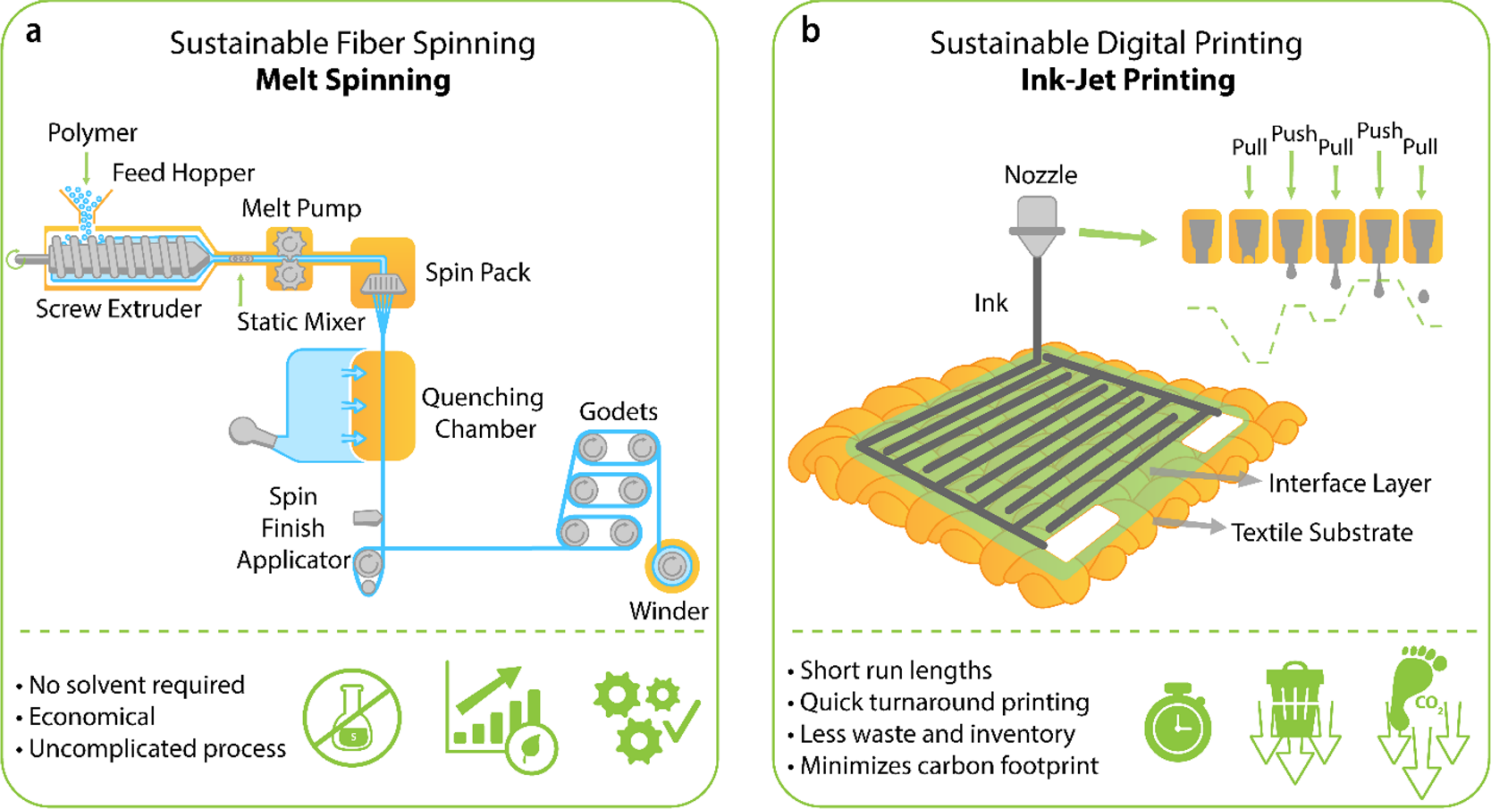
Sustainable manufacturing techniques: (a) fiber-spinning: melt-spinning.^284^ (b) Digital printing: inkjet printing.

Typically, man-made fibers are formed by “spinning” the polymer, which involves extruding the polymer in long lengths and dramatically increasing their aspect ratio, which results in the formation of polymeric fiber strands with small widths in the micron-scale or less. In dry-spinning process, polymers are dissolved in a solution that can be evaporated, whereas wet-spinning involves extruding the polymer/solvent mix into a second coagulation solvent. In contrast, melt-spinning is applied to polymers that can be effortlessly melted at a relatively low temperature. Melt-spinning is one of the simplest extrusion processes where no additional solvent is required, hence there is also no need for subsequent solvent removal. It is also considered one of the most economical and popular methods for polymer fiber manufacturing at industrial scales. The fiber-forming substance is melted just before to extrusion through the spinneret in melt-spinning and then consequently solidified by cooling into a cold-air quench duct (Figure 7a). Since no solvents are released in this process, there are no byproducts that have to be recycled. The extruded polymer cools and solidifies into continuous filaments, which is then drawn, twisted, further processed, and wound onto spools. From a wearable e-textile perspective, conductive nanomaterials such as graphene and CNTs at the fiber-spinning stage, can be integrated into the textile structure by adding straight into the polymer solution, which is later extruded together to generate conductive fibers or filament. Such a spinning method is suitable for producing the commodity fibers based on polyamide (nylon),^275^ PET,^276^ and PP fibers. In addition, in melt-spinning of e-textile fibers, PE and PP melts can also directly act as initiator to produce ultrafine fibers.^277^ Solvent-free melt-spinning, as compared to polymer-spinning in the solution phase, may present further opportunities for spinning without the danger of solvent residue in fibers, solvent evaporation into the atmosphere, or the high cost of solvent recycling. It also has the potential to have potential applications in the biomedical, tissue engineering, technical textiles, and filtration sectors.

Moreover, multicomponent melt-spinning takes advantage of capabilities not found in polymers alone, as beneficial mechanical, physical, or chemical properties of different materials can be merged in a single fiber, broadening the range of possible application domains.^278,279^ For instance, as the two polymers (e.g., PET/PP) can influence the thinning and solidification behaviors of each material along the spinning line, both components’ molecular structure development can influence each other.^280^ Several research groups have already investigated multicomponent and multifunctional electronic fibers for wearable electronics. Kapoor et al.^281^ presented a prototype multimodal and multifunctional sensing system constituted within a woven fabric structure with organized insulating and conductive portions. In addition, Lund et al.^282^ asserted melt-spinning of a piezoelectric bicomponent polymer fiber for developing a force sensor. That was eventually a carbon black (CB) and high-density polyethylene (HDPE) electrically conductive compound, respectively functioning as an inner electrode and core material, with poly(vinylidene fluoride) as the electroactive component. The piezoelectric effect of the fibers was comparable to that of commercial piezoelectric polymer films. Fink et al.^283^ developed a scalable preform drawing method for the preparation of multicomponent as well as multifunctional fibers. Multicomponent melt-spinning, which combines metals, insulators, and semiconductors within a single fiber strand, can then be used to produce single-material fibers with multifunctionality. Recognizing that traditional man-made polymer fibers may lack the geometry, compositional range, and feature sizes^283,284^ for extra functions, the expedition of the frontier of fiber functionality, multicomponent melt-spinning could be a productive area for future fiber production for wearable e-textiles.

Therefore, it can be summarized that the development of melt-spun fiber for wearable e-textiles are inextricably linked to fields of study such as polymer synthesis and processing, functionalization, multicomponent concepts, healthcare applications, as well as “frontier” electronics. Embedding multiple functionalities into a single fiber is still a massive obstacle, but advanced materials with enhanced rheological, electrical and other properties that retain their functional features when applied through melt-spinning should be further investigated. We anticipate that a single strand of fiber will exhibit extremely developed fiber architectures, both within its cross-section and length, to demonstrate electronic functionalities within a sustainable wearable e-textile sector using sustainable materials and fabrication approaches.

### Digital Printing

There are emerging trends around the use of manufacturing processes that are sustainable with low environmental impact. Sustainable manufacturing promotes processes that are energy/water efficient and use sustainable and recycled materials, eliminating hazardous substances wherever possible. In this regard, digital manufacturing offers many sustainability benefits, such as the minimal waste of materials, lower energy consumption, and less use of chemicals. For manufacturing sustainable and wearable e-textiles, the digital printing technique is attractive in being low-cost and utilizes existing machines but is still evolving in the use of robust, highly conductive inks.

Inkjet printing is currently at the forefront of flexible printed electronics. It offers an attractive route to low-cost, versatile and high-resolution multilayer printing (Figure 7b) of picoliter volume functional materials on generic textile substrates with multiple functionalities, united with a cutback in material waste and water utilization. Materials offering conductive, semiconductive and insulating properties are used in different combinations in inkjet printing, allowing the fabrication of various heterostructures (“designer devices”), which bring precisely tailored properties to have diverse functionalities and improved performance for applications. Inkjet printing of such devices will solve the reliability and manufacturing issues that current wearable devices face through the nonimpact and mask-less deposition of controlled quantities of materials in a rapid, precise, and reliable way. Inkjet printing will provide a sustainable alternative or complementary approach to the multicomponent melt-spinning of multimaterial fibers. However, the realization of fully inkjet-printed devices is extremely challenging on “rough and porous” textiles substrates with an intrinsic planar anisotropy of the general properties^198^ and continuously changing fiber morphology due to the orientation fibers or yarns and exchange of water molecules with surroundings, respectively.

In our previous study,^258^ all-inkjet-printed graphene-based wearable e-textiles were developed, which incorporated an organic surface pretreatment to allow printing of conductive (and potentially nonconductive and semiconductive structures) patterns via inkjet printing on rough and porous textile surfaces and reduced surface resistance by 3 orders of magnitude compared with the untreated textiles. This type of pretreatment acts as a receptor layer for water-based reduced graphene oxide (rGO)-based ink, which can then be dried at low temperatures (100 °C), lowering the risk of damaging heat-sensitive fabrics. However, the study was limited only to two-dimensional rGO-based inks with lower electrical conductivity, limiting their prospects for specific applications requiring higher electrical conductivity. In another study,^285^ we have already shown that graphene–Ag hybrid inks could well be inkjet-printed onto pretreated cotton fabrics, allowing us to create all-inkjet-printed, highly conductive, and cost-effective wearable e-textiles. However, the study involved the application of metallic Ag, which again suffers from environmental and biocompatibility issues. Recently, research on a screen-printed, flexible and machine washable electrically conductive e-textiles platform has been carried out to store energy and monitor brain activities (i.e., through EEG).^286^ Although screen printing is highly scalable, it suffers from poor print resolution, multistep processes for mesh preparation, and washings with material waste and water pollution.

Despite the clear potential of inkjet-printed wearable e-textiles, there have still only been a few attempts to produce wearable textiles of multimaterials via inkjet printing. Carey et al.^287^ reported the possibility of inkjet printing of graphene/h-BN field-effect heterojunctions for wearable and textile applications. However, the reported device was neither flexible, nor washable, and not entirely fabricated from inkjet printing. The surface pretreatment was coated on fabrics, which reduced the comfort and breathability of the device and limited the print resolutions. An initial study by Kim et al.^288^ on inkjet printing of multimaterials (metals: Ag nanoparticles, conductive polymer: PEDOT: PSS, and isolating inks: PVP) by a simple desktop inkjet printer showed promise for stretchable wearable e-textiles applications.

However, we anticipate that additional ground-breaking applications for inkjet-printed wearable e-textiles will emerge in the near future to satisfy the empirical demands for high-performance detection, data processing, computation, and display. The irresistible development of advanced inkjet printing technologies is accelerating the impact on wearable electronics textiles. For example, researchers have shown that inkjet printed material did not lose any electrical performance after more than 100 cycles.^289^ Compared to other processing routes, inkjet printing needs no special conditions or atmospheres to create such e-textiles and offers routine reliability in diverse applications such as energy harvesting, mainstream electronic gadgets or healthcare biomedical devices.

## Sustainability Assessment

The reasons for the growing interest in smart e-textiles and related applications can be ascribed to their great potential to establish innovative, rich as well as personalized material experiences. As such, metrics for describing e-textiles have been centered around their electronic performances and/or physical properties but less so on their sustainability. Evaluating the overall state of the e-textiles industry with sustainability aspects added to the criteria for assessment of product success and viability, it is apparent that these “smart” effect can be obtained with less environmentally harmful materials. However, it is often the case that the “environmentally-friendly alternatives” perform much worse in comparison to typically used fossil-fuel-based and/or non-biodegradable materials. As a result, research into how materials are perceived and how they can be used interchangeably to make different user experiences is ongoing,^290−292^ allowing manufacturers to be confident that the materials they are using are eco-friendly alternative solutions to fossil-fuel-based plastics. In this regard, a quantitative evaluation of various materials’ environmental performance over the entire wheel of life is needed alongside the existing metrics for a fair comparison of the sustainability of recently developed materials against other conventional materials for the same application. Specifically, we discuss the LCA as a vital system for selecting the best materials and processes as well as waste management techniques that promote sustainability and product quality.

### Life Cycle Analysis

LCA enables the quantification of environmental consequences of a product, service, or material, typically allowing better-informed decisions to be made in the design of that item or the formulation of specific policies.^294^ According to international standards, LCA is a quantitative environmental assessment method,^295,296^ which is widely used to investigate the potential environmental consequences of operations, goods, and services from the cradle to the grave.^297,298^ It includes the acquisition of raw materials, the manufacturing processes that lead to products, the transportation processes, the product use phase, and the EoL stages. The impact on the environment of a product can be analyzed and contrasted with other products or possible alternative solutions using the LCA methodology. Generally, LCA evaluation starts with setting the goal and scope, followed by inventory, impact and improvement assessment. Materials and energy from the inventory stage are used in processes ranging from raw material extraction to manufacturing, distribution, transportation, use as well as maintenance, disposal, and recycling. These are then benchmarked within the framework of their impacts on factors such as global warming potential, air, water, soil pollution, ecotoxicity, and resource depletion. International standards (ISO 14040 and others) place strict control on what qualifies as an LCA and which items are permissible.^294^

**Figure 8:**
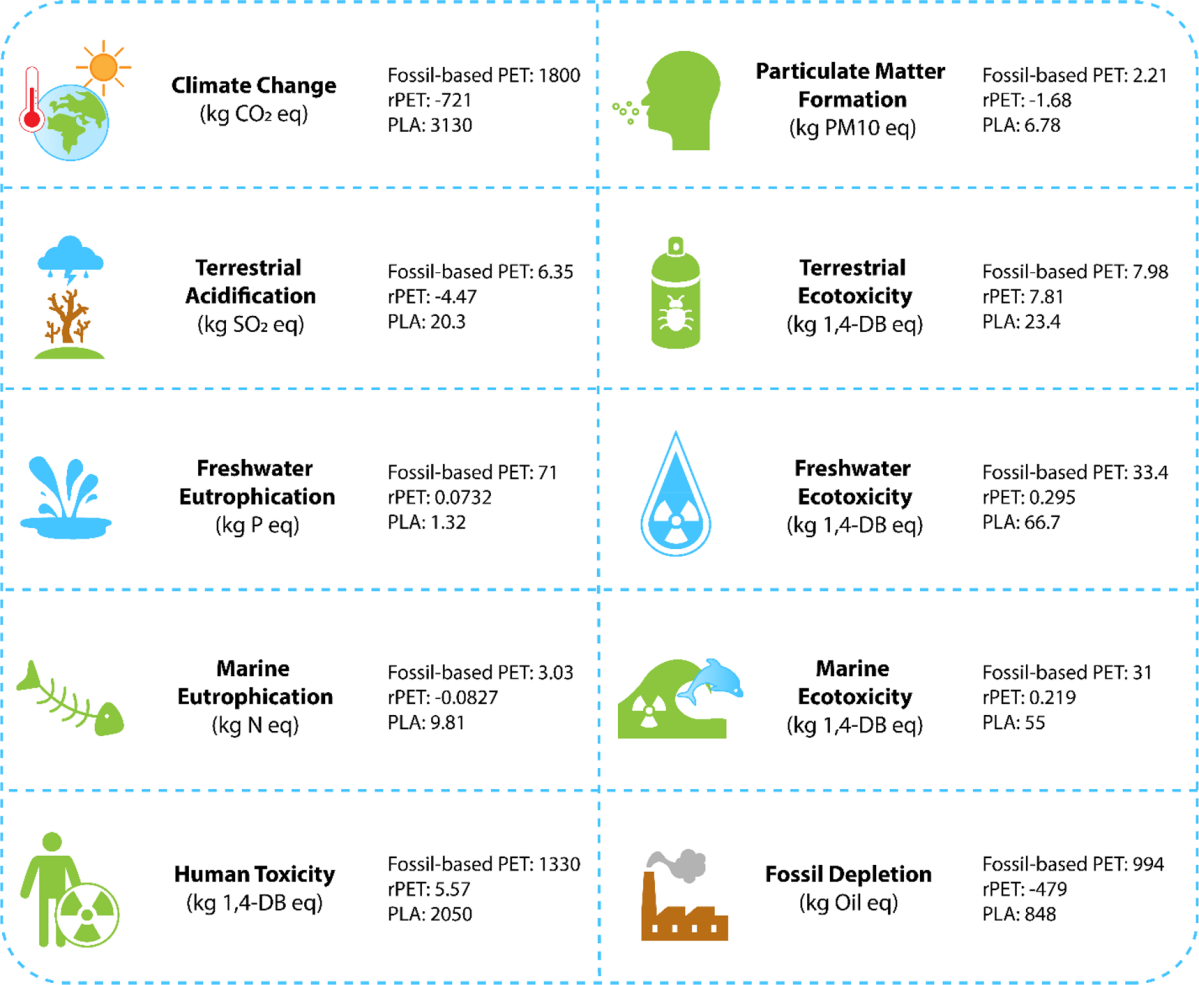
Environmental impact comparison among fossil-fuel-based PET, rPET, and PLA.^293^

There have been a few environmental assessment studies on products with similar characteristics to smart textiles, such as an LCA of a printed antenna^299^ and an environmental LCA of a prospective nanosilver T-shirt.^300^ LCAs of traditional textile products^301,302^ based on cotton, polyester (PET), nylon, acrylic, and elastane without smart functionality are readily available, but so far, no LCA studies of smart textile products have been established. As a result, despite extensive research on LCAs of individual materials, it is not possible to draw conclusions about future LCA results of smart textile products, as the impact of the combination of textile and electronic materials in one product is still unknown. For instance, in a study,^297^ two promising sustainable alternatives to PET plastics, 100% rPET and bio-based plastics based on PLA, were analyzed as a potential fiber base material for wearable e-textiles. The comparison of environmental impact among fossil-fuel-based PET, rPET, and PLA on different parameters, such as climate change and toxicity, is illustrated in Figure 8. Both recycled and bio-based plastics are frequently criticized because of their limited environmental benefits compared to fossil-fuel-based plastics. Nevertheless, they are now a sustainable choice of consideration as promising alternatives to conventional fossil-fuel-based plastics. rPET offers essential ecological benefits compared to traditional fossil-fuel-based PET during the manufacturing and end-of-life management stages. Additionally, PLA shows clear environmental advantages over PET, irrespective of freshwater eutrophication and human toxicity. Hence, a lifecycle perspective on the consequences of design choices, material selection and manufacturing techniques can now guide the implementation of sustainable manufacturing.

### Biodegradability

**Figure 9:**
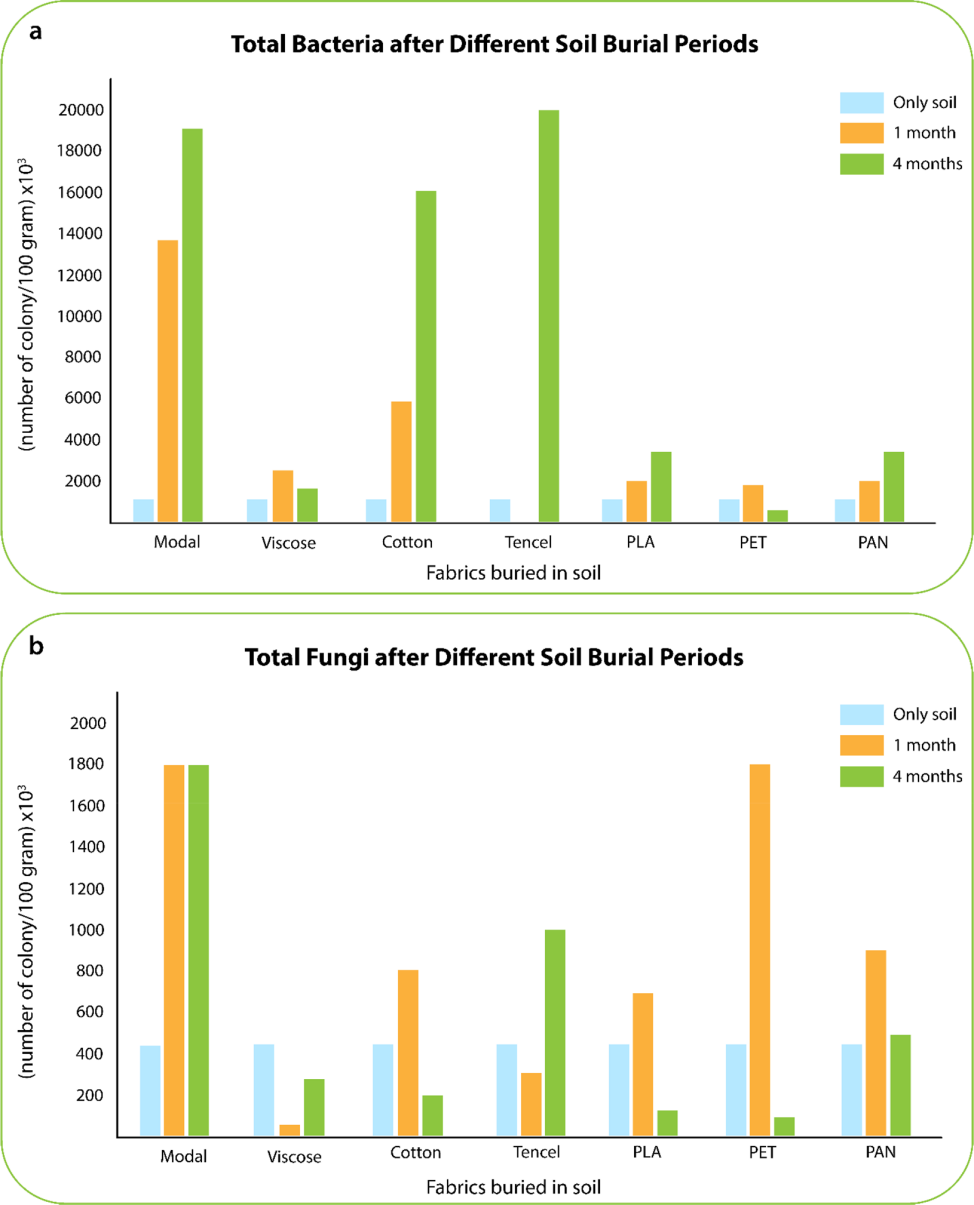
(a) Influence of burial interval on soil bacteria total values. (b) Influence of burial interval on soil sample total fungi values.^306^

The capability of a substance to decompose after interactions with biological elements is known as biodegradability. The conditions of the environment, including matrix composition and temperature, will also influence the rate at which a product will breakdown/decompose.^303^ During biodegradation, numerous microorganisms such as fungi, bacteria, worms, as well as many other species attack the materials,^304^ for instance, during soil burial tests.^305^ Therefore, many studies examined the total bacteria and fungi in soil samples to ascertain the biodegradation behavior patterns of textile materials.^306^ Unmodified natural fabrics such as cotton, hemp, linen or bamboo will often decompose more quickly than synthetic textiles.^307^ Due to their structure, synthetic fabrics like nylon, acrylic and Lycra will naturally take more time to biodegrade.^308^ Sular and Devrim^306^ concluded in their study that a 4 month burial interval is sufficient for assessing the biodegradation behavior of cellulosic fibers, while periods longer than 4 months are required to properly assess the biodegradation behavior of synthetic fibers (Figure 9). The biodegradability of textiles is altered according to the chemistry added/used during the manufacture of the material or the product lifecycle (regardless of whether the material started as a natural or synthetic fiber).^307^ In addition, not all natural materials may be defined as “sustainable” due to the nature of the techniques used by researchers for assessing biopolymer biodegradation evaluations such as CO_2_ generation, molecular weight decrease, residual weight analysis or weight loss measurement, and mathematical modeling.^309,310^ Biodegradation of bio-based and biodegradable polymer PLA under composting conditions involves two pathways, that is, hydrolytic degradation followed by biodegradation (microbial assimilation).^311^ The abiotic degradation^310^ process of PLA-based biocomposites at composting temperatures is rarely discussed.

Besides bio-based and biodegradable fiber materials PLA, we have discussed type I conductive polymers in previous sections, which can be partially disintegrated, and type II conductive polymers, which can be dissolved aided dopants. However, for a product to breakdown and decompose fully into the surrounding environment, both disintegration and biodegradation must occur. Disintegration is the physical process of material breakdown. In contrast, biodegradability is the breakdown of the product back into fundamental components such as water, biomass, and gas through a chemical reaction. During biodegradability testing, the evolution of CO_2_ or O_2_ is measured to determine the rate of biodegradation. Establishing how a product or material disintegrates and biodegrades is achievable through testing.^312^ The standard test methods for determining the anaerobic biodegradation of plastic materials under accelerated landfill conditions and simulated laboratory conditions, respectively, are ASTM D5526 and ASTM D5511.^313^ ISO 20200:2015 disintegration testing can evaluate materials for their propensity to disintegrate in a compost environment. ISO 20136 Biodegradability testing will evaluate whether the product can completely break down to a state that mimics the natural environment. This type of testing measures the capability of a product to be degraded by microorganisms, where a measure of CO_2_ produced is used to assess the extent of biodegradation.^306,314^

### Recyclability

Recyclability is the capability of a product to be reused at the end of its multiple lifetimes to reduce waste, pollution, and resource use. Recycling, on the other hand, is the process of converting waste materials into reusable materials and byproduct objects, for recycling to be economical, there must be an ample supply of the homogeneous material, a supply chain to collect and process those materials and a market-driven demand. When considering recycling products in general, fundamental factors need to be checked, such as whether the recycled material is cheaper to use than the primary virgin material. First, we must establish whether using recycled materials instead of virgin materials to make a product can save energy. Then, we should confirm if the recycled material needs to be free of contamination and whether this material can economically meet the same quality and performance standards.

One report found that only 9% of all plastics are recycled,^315^ where only plastic PET and HDPE bottles and containers meet the minimal legal standards to be labeled as recyclable. Other materials typically end up in landfills or incinerators, polluting the environment.^316^ An important feature of PET is that it has a well-established recycling process and can be recycled several times as rPET. Its excellent physical properties enable the manufacture of lightweight containers, which reduces the use of natural resources. Plastic is collected, cleaned, and remade into products during the recycling process. As the reuse and recycling of plastics reduce carbon emissions by at least 24%, rPET can be considered a more sustainable alternative to PET (Table 6). Postconsumer PET bottles or rPET can be mechanically recycled into sheets, films, and fibers that are used for sacking, insulations strapping packaging, and floor coverings.^317^ It is also possible to recover feedstock materials from PET or generate fresh materials through chemical recycling, which can then be reused as fiber materials in wearable e-textiles. Acids, methanol, glycols, ammonia, and amines are used in the chemical recycling of PET, each chemical treatment (hydrolysis, methanolysis, glycolysis, ammonolysis, and aminolysis, respectively) having its individual relative merits.^318,319^ For sustainable development, chemical recycling is the simplest route to obtaining the raw materials from which PET is initially created. The ultimate goal of chemical recycling of PET is to increase monomer yield while reducing reaction time and conducting the chemical reaction under mild conditions.^320^ Ongoing R&D has brought about significant improvements in the chemical recycling processes.^321^ ISO 15270:2008 directs the progress of standards and guidelines for the recycling and recovery of plastic waste.^322^ The standard specifies the various options for recovering plastic waste from pre- and postconsumer sources.

**Table 6:** Sustainability Profile of Fibrous Polymers Used Fiber in Wearable E-Textiles

polymers	raw materials	biodegradability	composting	recyclability	energy consumption	carbon footprint	environmentally friendly
rPET	postconsumer PET bottles	nonbiodegradable	noncompostable	can be recycled several times from PET which stands as rPET	35 MJ/kg^324^	0.45 kg CO_2_ equivalent per kilogram of rPET^324^	rPET reduces energy consumption 79% and greenhouse gas emissions 67%
PLA	100% renewable source/bio-based: corn	biodegradable by microorganism	industrial-scale composting needed which is not enough in facilities, i.e., conditionally compostable	other plastic in resin identification, sometimes recyclable: but not in bulk scale	58.9 MJ/kg^325^	500 g CO_2_/kg^325^	production uses 65% less energy than conventional virgin plastics; bioplastics are safe as food packaging materials, though they are not microwaveable

In the long term, the recycling of nonrenewable materials, like postconsumer PET bottles, may decrease environmental pollution and protect natural ecosystems, but it is important we ensure that *every* recycling operation and *every* material is sustainable and beneficial for our environment. Recycling is a prolonged and circular process, so it is vital to understand the dynamics of the evolving process. With climate change being a global concern, reducing greenhouse indicator gas emissions is key in securing environmental sustainability and ensuring the future of the world’s economy and ecology.^323^ If recycling can use less energy to make the final product than the primary usage of virgin materials, then the energy generation costs are similarly reduced. Thus, the optimized supply chain leads to less pollution, lower cost, reduced environmental damage, and thus better environmental sustainability. Further, by redirecting from landfill and minimizing the discharge of incineration gases such as carbon dioxide, carbon monoxide, and other toxic gases,^323^ we can directly influence global warming and climate change. Therefore, recycling is a vital component of sustainability in consideration of the global environment.

## Future Directions and Outlook

Many fibers, such as cotton and silk, are biodegradable. However, their processing is increasingly unattractive and appears to be unsustainable. The use of alternative biodegradable and recycled materials again cannot safeguard against all hazards for making a sustainable wearable e-textile and may not be able to achieve the target mechanical and electrical properties alone. Fabrication techniques, end-of-life management of electronic components and materials, working environment, duration of exposure, type of associated hazards, atmospheric conditions and many other factors are related to selecting appropriate materials and techniques for sustainability.

First, considering the available “sustainable” fibers that offer bio-based, biodegradability and recyclability, materials such as PLA, rPET, and rPP may fulfill the sustainability criteria outlined in Figure 1b. Moreover, there have also been significant progress in the further development of sustainable biopolymer fibers modified with graphene or other conductive 2D materials. This modification enhances the mechanical and electrical properties of the fibers, facilitating their use in wearable electronics. This advancement represents only one of several recently reported functional materials that could be used for e-textiles, each one outperforming the previous in any given aspect. Even with this rapid development, there remains a challenge in directly comparing the performances of one type of electronic fibers/yarns/fabrics with another absence of consistent technical and testing standards. At present, the characterization system used in the evaluation of biodegradability parameters for electronic fibers/yarns/fabrics are inconsistent across different reports, making it difficult to establish a balanced comparison between different samples and processes. Further, in recognizing that the study of electronic fibers/yarns/fabrics is interdisciplinary by nature, joint cooperation by scientists across the disciplines will become increasingly necessary to establish environmentally conscious, factually consistent and simple evaluation standards. By doing so, we will increase the potential for commercializing electronic fibers/yarns/fabrics as well as incorporating them in flexible and wearable electronic textiles for immediate uses in the fields of human health monitoring and various other smart devices.

**Figure 10:**
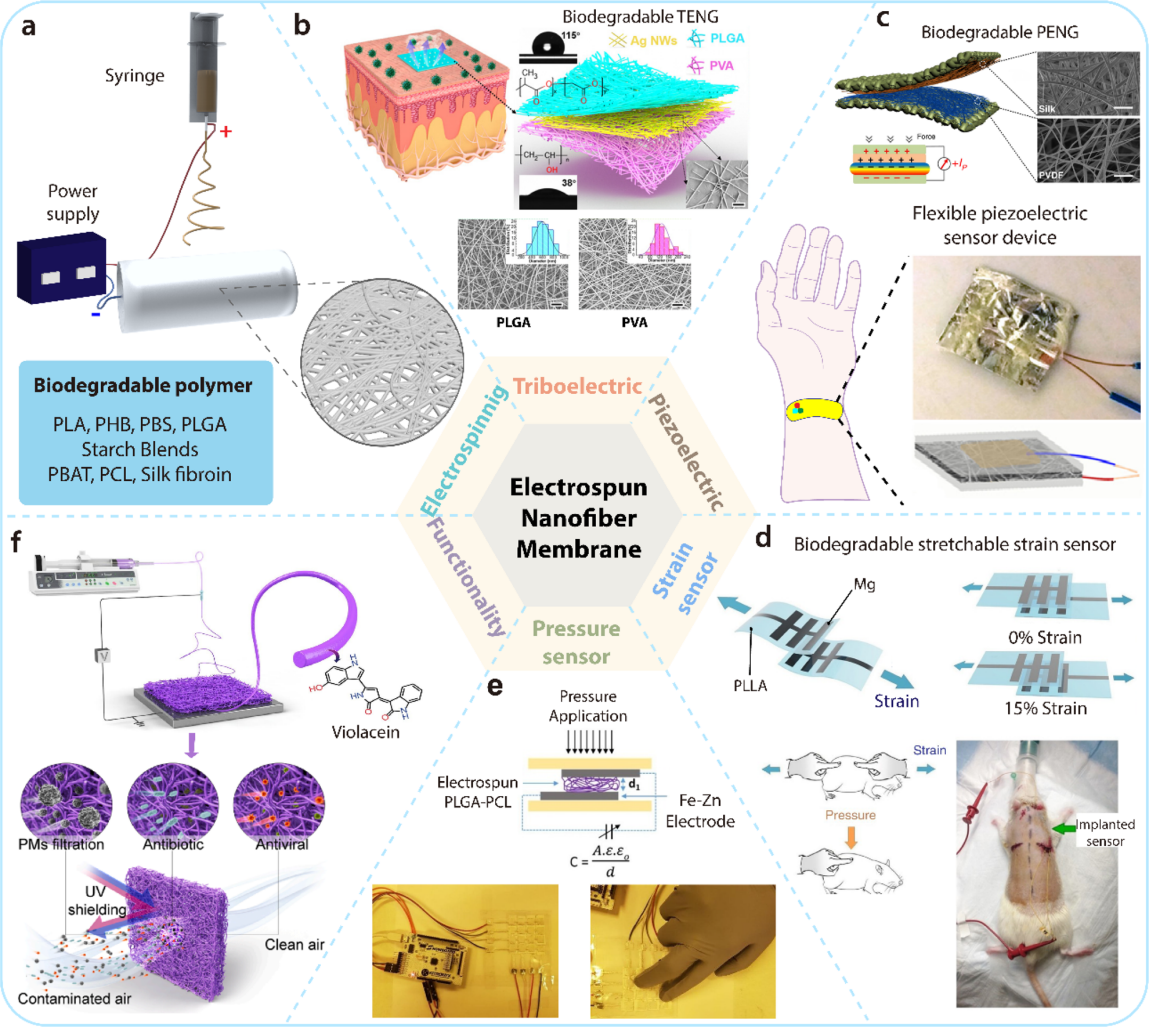
Applications of electrospun nanofiber membranes. (a) Electrospun nanofiber membrane using various biodegradable polymers. (b) Schematic illustration of the 3D network structure of the all-nanofiber TENG-based e-skin and the application scenario for the e-skin can be quickly and conformally bonded to the epidermis and is porous, biodegradable, and antimicrobial.^326^ (c) Schematic illustration of an all-fiber hybrid triboelectric nanogenerator comprises electrodes (conductive fabric) and a triboelectric pair made of electrospun silk and PVDF nanofibers and schematic illustration of flexible sensor assembling and recording of radial artery pulse signals of a candidate. Reprinted with permission from ref (327). Copyright 2020 American Chemical Society.(d) Schematic illustration of biodegradable strain sensor. Strain sensing: When strain is applied, the two thin-film comb electrodes move about one another, causing the capacitance to change. As the strain on tendons in real life is less than 10%, the strain detecting range of 0 to 15% was chosen. Reprinted with permission from ref (332). Copyright 2018 Springer Nature. (e) Schematic illustration of a highly sensitive biodegradable pressure sensor and a commercially available pressure mapping platform coupled to a 4 by 4 array produced using a dielectric membrane and application of pressure. Reprinted with permission from ref (331). Copyright 2019 Elsevier. (f) Schematic illustration of the fabrication and properties of violacein-embedded nanofiber filter. Reprinted with permission from ref (328). Copyright 2022 Elsevier.

Second, the development of environmentally friendly fabrication methods for e-textiles requires actively assessing and minimizing the environmental consequences of the manufacturing, usage as well as recycling of e-textiles.^293^ Producing textiles with robust mechanical, thermal, electrical and antimicrobial properties are achievable, but longevity requires alternative approaches focused on avoiding overconsumption of materials and using less harmful solvents, via inkjet printing and melt-spinning methods, respectively. To achieve a robust manufacturing technology, we need to devise simple solutions to solve the issues with inkjet nozzle clogging for a durable printing method and the improved longevity with all sorts of inks is required. Another eco-friendly direction is to reduce the number of polymer materials used in the manufacturing of e-textiles. In this regard, we may consider the use of electrospinning as a viable alternative technique to fabricate biodegradable polymeric nanofiber membranes for use in flexible/wearable electronics applications (Figure 10). Based on their structural merits of high surface area, ease of manufacturing, and design flexibility, electrospun biodegradable membranes have shown to be promising for providing higher functionalities than conventional melt-spinning and inkjet processes.^326,327,329,330^ One of the most studied materials are biodegradable polymers with triboelectric and piezoelectric properties, which induce the redistribution of charges in a controllable manner depending on how the membrane is mechanically deformed. There are several examples in the literature that demonstrate the successful electrospinning synthesis of biodegradable tribo-/piezoelectric polymers, such as poly(lactic-*co*-glycolic acid) (PLGA) as well as silk fibroin, which could be used in energy harvesting and sensing applications.^326,327,329,330^ In fact, electrospun biodegradable membranes have also been utilized as the essential component in “electronic skin” devices, which are biocompatible sensor arrays that detect changes in multiple physicochemical parameters for providing on-site health monitoring and diagnostics.^331−333^ Moreover, electrospinning supports the incorporation of functional molecules in the nanofiber membrane itself, which is beneficial for wearable textiles because it provides additional functionality.^328,334^ A recent report by Lee et al. synthesized nanofiber membranes functionalized with violacein, a naturally occurring violet-colored pigment, by electrospinning a polymer solution containing the violacein molecules.^328,334^ As a result, in addition to the membrane’s ability to filter out particulate matter (PM), the violacein-embedded membrane showed antibacterial and antiviral properties as well as UV-shielding performance. Though this polymer is not strictly biodegradable, we highlight this case study as an example of the versatility of electrospinning in adding desired functionalities to fiber membranes. Altogether, these examples demonstrate the versatility of electrospinning as a facile approach to incorporate various functionalities into the nanofiber membrane and therefore need to be considered in the design of future e-textiles.

Similarly, manufacturers should ensure that diverse data paths, parallel lines, and fool proof circuit design are used, wearable e-textiles still focus on the minimal use of materials, such as nanomaterials. Graphene has the potential to enhance the performance of different materials and alleviate their carbon footprint. Recently, it has been reported that washable, durable, and flexible graphene-based wearable e-textiles are highly scalable, cost-effective, and potentially more environmentally friendly than existing metals-based technologies.^1,258,335,336^ Additionally, graphene and other 2D materials have sparked considerable interest in flexible and wearable electronics applications because of their electrical, mechanical^73,337^ and other performance properties. Those very attributes may be employed in heterostructures and furthermore.^338^ As more of a result, integrated graphene-based wearable e-textiles may be capable of dealing with both sustainability and current technical challenges associated with various applications, such as activity monitoring, early detection of highly transmissible diseases, and securing the health and well-being of frontline workers.

Moreover, fiber-based wearable electronic devices integrated into garments can offer comfort, breathability, lightweight, flexibility, and extensibility. They are engineered platforms interfacing and linking the environment, electronic devices and the human body. It also provides current possibilities for incorporating sustainable materials into energy storage devices, activity monitoring, disassembling wearable textile circuits for durability, and more extended usage, by setting up electronic hardware on the textile surface quickly and in a removable way. The quality, disposability, wearability, repairability, functionality, technological and aesthetic obsolescence will improve wearable e-textiles. Designing a sustainable wearable requires the integration of materials design, comfort, durability, recyclability and efficient removal of nonrecyclable electrical elements to enable a simple, cost-effective circularity. It is critically important to develop efficient technologies to make sustainable, breathable, flexible, and large-scale electronic fibers while guaranteeing that the performances of the electronics meet the demands of commercial application potentials of sustainable wearable e-textiles.

At present, researchers are producing at the laboratory scale sustainable electronic fibers and mainly weaving into electronic textiles by hand. Developing innovative and efficient machine fabrication technologies to form wearable e-textiles to promote large-scale fabrication is vital. In addition to this challenge, integrating multifunctions into wearable e-textiles is one of the significant challenges due to the conflict between different functions. In addition to the processes discussed above, including sensing, energy harvesting, and energy storage, the integration of fiber/textile-based circuits, information acquisition components, personal data security functions, and computing units has been little explored.

Lastly, the user must have sufficient information to care for the textile at home with minimal environmental impact. As wearable electronics combined with textiles (e-textiles) move from the bespoke maker into production and the mass market, issues concerning the laundering, disposal and waste electronics will increasingly arise. The sustainability of e-textiles, therefore, is determined at the start of the design process. We must consider several questions, such as whether the e-textile can be disassembled and recycled with ease, whether the e-textile can be repaired in a simple manner and whether the e-textile incorporates the necessary circuitry to support off-site software updates over wireless communications. Even though different alternatives have been reported to improve the performance of wearable e-textiles, such as the introduction of more sustainable conductive materials or the design of device structures, a balance between their electrical and mechanical properties has not been achieved. Furthermore, the cycling stability and washability of electronic fibers/textiles must be improved in order to advance energy harvesting, utilization, and storage.

By addressing these challenges in the early stage development of mass production wearable e-textiles, we can expect to minimize the negative environmental impacts. Not only are these efforts beneficial at the current stage, where the e-textiles technology remains limited to highly specialized applications, but they are also expected to expedite its transition from the laboratory to the marketplace as mass commodity. Amidst this e-textile evolution, researchers will no doubt attempt to take advantage of high-performance and multifunctional materials by incorporating varying amounts of functional materials, including not only graphene and other 2D materials but also eco-friendly functional fillers, within a fibrous, bio-based, biodegradable, and recyclable textile framework.^343^ In achieving this, we must deliver a sustainable, safe environment with associated social and economic benefits over the entire product life cycle. Thus, “3R” rules of environmental sustainability,^344^ i.e., “reduce, reuse, and recycle,” can be achieved by extracting raw materials up to the final disposal.

**Figure 11:**
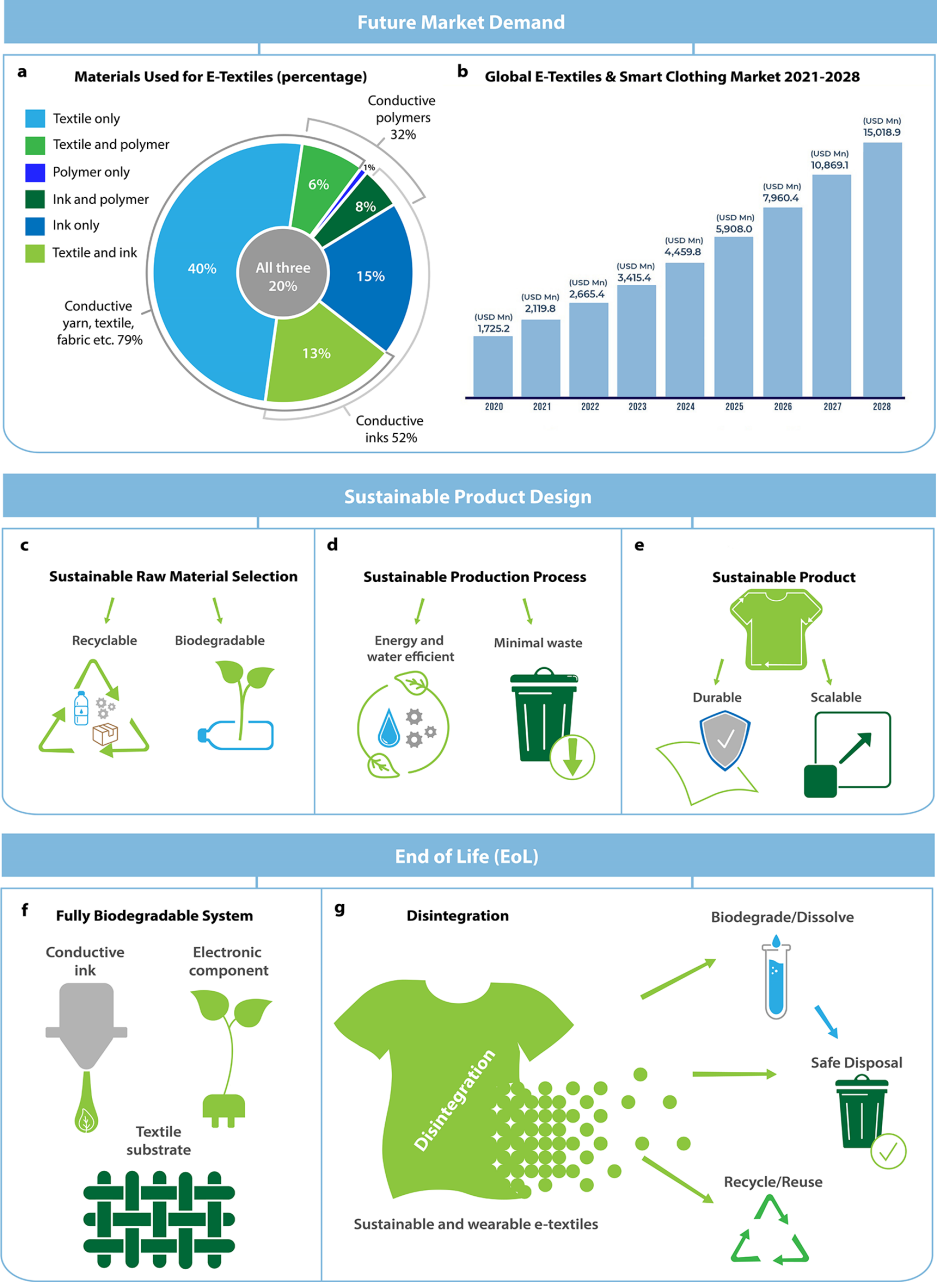
Toward environmental sustainability of wearable e-textiles. Future market demand: (a) Materials used for e-textile players 2019–2029,^339,340^ (b) global e-textiles and smart clothing market 2021–2028.^341,342^ Sustainable product design: (c) Sustainable raw materials selection, (d) sustainable production process, (e) sustainable product. End of life (EoL): (f) fully biodegradable system, (g) disintegration.

In summary, the market for smart wearable electronics offers great potential for sustainable materials and it is anticipated that like traditional commodity materials, wearable textiles with distinctive forms and functionalities will become a part of all of our lives in the future. They will not simply meet the requirements of everyday wear, but they will also serve the arising fields of personalized healthcare and human-machine interface, which will revolutionize our way of life. Even though some optimistic advancements have been made in certain areas, many diverse challenges remain. We believe that the continuous endeavors of researchers from different fields will further improve sustainable, wearable electronic textile design, functionality and performance, thus promoting the development of sustainable materials and manufacturing techniques toward a viable commercial future (Figure 11).

## Vocabulary

**Wearable electronics**: electronic devices that are continuously worn by an individual, much like garments, to provide intelligent assistance that enhances memory, intellect, originality, interaction, and physiological senses
**Electronic textiles**: also known as e-textiles, are fabrics that can be embedded with electronic components such as batteries, lights, sensors, and microcontrollers
**Smart textiles**: materials and structures that sense and respond to environmental conditions or stimuli such as thermal, mechanical, electrical, chemical, magnetic, or other sources
**Sustainability**: the ability to cause little or no environmental damage and thus to sustain for a long time
**Biodegradability**: the ability of living organisms to degrade organic materials biologically to base substances such as water, carbon dioxide, methane, basic elements, and biomass
**Composting**: a man-made process in which biodegradation occurs under specific conditions
**Recycling**: the transformation of waste materials into reusable materials and objects
